# Transcriptomic uniqueness and commonality of the ion channels and transporters in the four heart chambers

**DOI:** 10.1038/s41598-021-82383-1

**Published:** 2021-02-02

**Authors:** Sanda Iacobas, Bogdan Amuzescu, Dumitru A. Iacobas

**Affiliations:** 1 Department of Pathology, New York Medical College, Valhalla, NY 10595 USA; 2 Department Biophysics and Physiology, Faculty of Biology, University of Bucharest, Bucharest, Romania; 3 Personalized Genomics Laboratory, Center for Computational Systems Biology, Roy G. Perry College of Engineering, Prairie View A&M University, Prairie View, TX 77446 USA; 4 DP Purpura Department of Neuroscience, Albert Einstein College of Medicine, New York, NY 10461 USA

**Keywords:** Cardiology, Gene expression

## Abstract

Myocardium transcriptomes of left and right atria and ventricles from four adult male C57Bl/6j mice were profiled with Agilent microarrays to identify the differences responsible for the distinct functional roles of the four heart chambers. Female mice were not investigated owing to their transcriptome dependence on the estrous cycle phase. Out of the quantified 16,886 unigenes, 15.76% on the left side and 16.5% on the right side exhibited differential expression between the atrium and the ventricle, while 5.8% of genes were differently expressed between the two atria and only 1.2% between the two ventricles. The study revealed also chamber differences in gene expression control and coordination. We analyzed ion channels and transporters, and genes within the cardiac muscle contraction, oxidative phosphorylation, glycolysis/gluconeogenesis, calcium and adrenergic signaling pathways. Interestingly, while expression of *Ank2* oscillates in phase with all 27 quantified binding partners in the left ventricle, the percentage of in-phase oscillating partners of *Ank2* is 15% and 37% in the left and right atria and 74% in the right ventricle. The analysis indicated high interventricular synchrony of the ion channels expressions and the substantially lower synchrony between the two atria and between the atrium and the ventricle from the same side.

## Introduction

Starting with crocodilians, the heart pumps the blood through the pulmonary circulation and the systemic circulation by the coordinated rhythmic contractions of its upper left and right atria (LA, RA) and lower left and right ventricles (LV, RV). Each of the four heart chambers has a well-defined role in circulation and the adequate morphology but how their distinct functions and buildups are determined by the transcriptomic profiles is still not completely understood.

There are important left–right differences in the mean arterial pressure between systemic and pulmonary circulation measured invasively in mice. The mean RV systolic pressure of 16.3 mm Hg^1^ vs. mean LV systolic pressure of 96.2 mm Hg^1^ leads to a five times greater LV workload compared to RV. There are also LV-RV differences in intraparietal tension, oxygen consumption and metabolic stress, resulting in higher susceptibility to oxidative stress, reduced angiogenic response and higher likelihood of activation of cell death pathways^2^.

A recent publication^3^ compared the regulation of genes involved in atrial fibrillation (AF) from paired human left and right atrial appendages of healthy and AF patients, concluding that there are “different mechanisms for development, support and remodeling of AF within the left and right atria”. Other authors compared the gene expressions from the left and right aortic arches of the chick embryo^4^. Direct comparison of gene expression profiles among the heart chambers improves our understanding of the heart embryogenesis^5^, signaling pathways^6^, transcriptional enhancers and gene regulatory networks^7,8^, involvement of miRNAs^9^, epigenetic reprogramming mechanisms^10^ etc.

We previously reported significant sex differences in the expression and networking of mouse heart rhythm determinant genes (HRD), with males having higher expression in atria than in ventricles and females higher expression in ventricles than in atria^11^. We have reported also sex differences in subcellular localization of HRD proteins and that both expression and localization of HRD proteins in female heart change during the estrous cycle^12^. Since female transcriptome depends on the estrogen level^13^ (uncontrollable variable) and phase of the estrous cycle (requiring synchronized animals in each of the four phases), the present research was restricted to males. However, there are studies of chamber-specific gene expression profiles in female mice^14^ but without mentioning whether the animals were in the same or different cycle phase.

A number of molecular mechanisms have been proposed to explain the left–right asymmetry at the heart level and in the internal organization of the amniote embryo. Starting from Kartagener’s syndrome, a rare autosomal recessive disorder characterized by primary ciliary dyskinesia associated with *situs inversus*^15^, researchers have proposed as primary left–right asymmetry determinant a mechanosensitive detection mechanism of unidirectional yolk sac fluid movement. One interesting hypothesis emphasizes the role of *Pkd2* (polycystin 2, transient receptor potential cation channel), in sensing the leftward flow of fluid in the yolk sack produced by unidirectional rotation of cilia on the endodermic surface of the Hensen node^15^. The yolk sac fluid movement is generated by cilia rotation on the endoderm side of the primitive node, involving TRPP2 ion channels belonging to the polycystin subfamily of transient receptor potential (TRP), activation of non-canonical Hedgehog pathway and asymmetrical calcium signaling^16,17^. Another rare clinical condition, the Holt-Oram syndrome, associated with congenital heart defects^18^, has led to an elegant demonstration using differential gene expression methods of the rheostatic control exerted by the transcriptional activator *Tbx5* in interventricular septum formation and ventricular patterning. Other researchers have evidenced the role played by differential left–right expression of Nodal and bone morphogenetic (BMP) signaling pathways along the lateral plate mesoderm, leading to asymmetrical activation of transcription factors Pitx2 and Prrx1 and subsequent heart laterality in vertebrates by asymmetrical epithelial-to-mesenchymal transition^19^. Whole-cell patch-clamp studies on isolated cardiomyocytes have demonstrated the role of Notch1 signaling pathway in achievement of a mature shortened triangular ventricular action potential (AP) phenotype and the role played by various voltage-dependent K^+^ channels (Kv) subunits and interacting proteins like KChIP2^20^.

The ionic channels and transporters (ICTs) play fundamental roles in the heart physiology and their deficient expression and/or configuration are responsible for a number of severe cardiac channelopathies^21^ that dysregulate the rhythm, synchrony and strength of the heart contractions. Channelopathy symptoms depend specifically on what ICT was altered and how and how much its function was affected. Major ICT-related heart diseases include: Brugada syndrome^22^, long^23^ and short^24^ QT syndromes, and catecholaminergic polymorphic ventricular tachycardia^25^.

The present study, carried out in compliance with the ARRIVE (animal research reporting of in vivo experiments) guidelines (https://arriveguidelines.org) aimed to assess the transcriptomic commonality and uniqueness in myocardium samples from the four heart chambers of adult C57Bl/6j male mice.

Owing to their critical role in the heart physiology, a particular emphasis was placed on expression coordination among the ICT genes in each chamber and the degree of their expression synchrony between adjacent chambers. We have explored a number of functional pathways, addressing the differences in expression level, control and coordination between the two atria, the two ventricles, and between the atrium and the ventricle on the same side.

A special attention was given to the expression coordination of *Ank2* (encoding Ankyrin-B), a major player in cardiac physiology, with its potential binding partners^26^ in each heart chamber. The coordination analysis with *Ank2* partners was extended to *Ctnnb1* (catenin (cadherin associated protein), beta 1),* Hspa5* (heat shock protein 5) and *Mapk1* (mitogen-activated protein kinase 1) which together with *Ank2* were identified as hub-bottleneck genes in atrial fibrillation^27^.

We used the Genomic Fabric Paradigm (GFP) that goes beyond the traditional transcriptomic analysis limited to the average expression level (AVE) of individual genes by considering also their relative expression variability (REV) and correlation (COR) with each other gene^28^. REV informs about the strength of the cellular homeostatic control to confine the expression fluctuations into narrow intervals. COR analysis responds to the “Principle of Transcriptomic Stoichiometry”^29^, a generalization of the “Law of Multiple Proportions” from chemistry, requiring genes to be expressed in certain proportions to optimize the functional pathways. Thus, GFP provided the most (theoretically possible) comprehensive characterization of the topology of each heart chamber transcriptome and their differences. Moreover, in each chamber, we have also established the gene hierarchy according to their Gene Commanding Height (GCH) score and identified the corresponding Gene Master Regulator (GMR)^29^. GMR is the gene whose highly controlled expression level by the cellular homeostatic mechanisms regulates most functional pathways.

Gene Ontology Consortium (www.geneontology.org) and Kyoto Encyclopedia for Genes and Genomes (https://www.kegg.jp) were used to select the genes encoding ion channels and transporters (ICT, 199 genes) and genes involved in the following pathways: adrenergic signaling in cardiomyocytes (ASC, mmu04261, 104 genes), calcium signaling (CAS, mmu04020, 121 genes), cardiac muscle contraction (CMC, mmu04260, 65 genes), glycolysis/gluconeogenesis (GLY, mmu00010, 50 genes) and oxidative phosphorylation (OPH, mmu00190, 111 genes).

## Results

### Expression level, variability and coordination are independent features

Raw and processed microarray data were deposited as GSE45339 and GSE45348 at https://www.ncbi.nlm.nih.gov/geo/. After grouping the spots probing redundantly the same transcript, we arrived at 16,886 unigenes that were adequately quantified in each of the four chambers from each of the four mice (total 16 samples). As designed, the experiment with four biological replicas provided three independent measures for each gene in each chamber: AVE, REV and expression COR with each other gene^28^. Thus, in addition to the 16,886 expression levels of the quantified unigenes in each chamber, we analyzed also 16,866 variabilities + 142,222,545 (16,866*16,865/2) expression correlations of distinct gene pairs, a tremendous increase of the transcriptomic data. We have also analyzed the expression synchrony of all genes in any pair of adjacent chambers, i.e. 67,464 (= 16,866*4) pairs.

**Figure 1:**
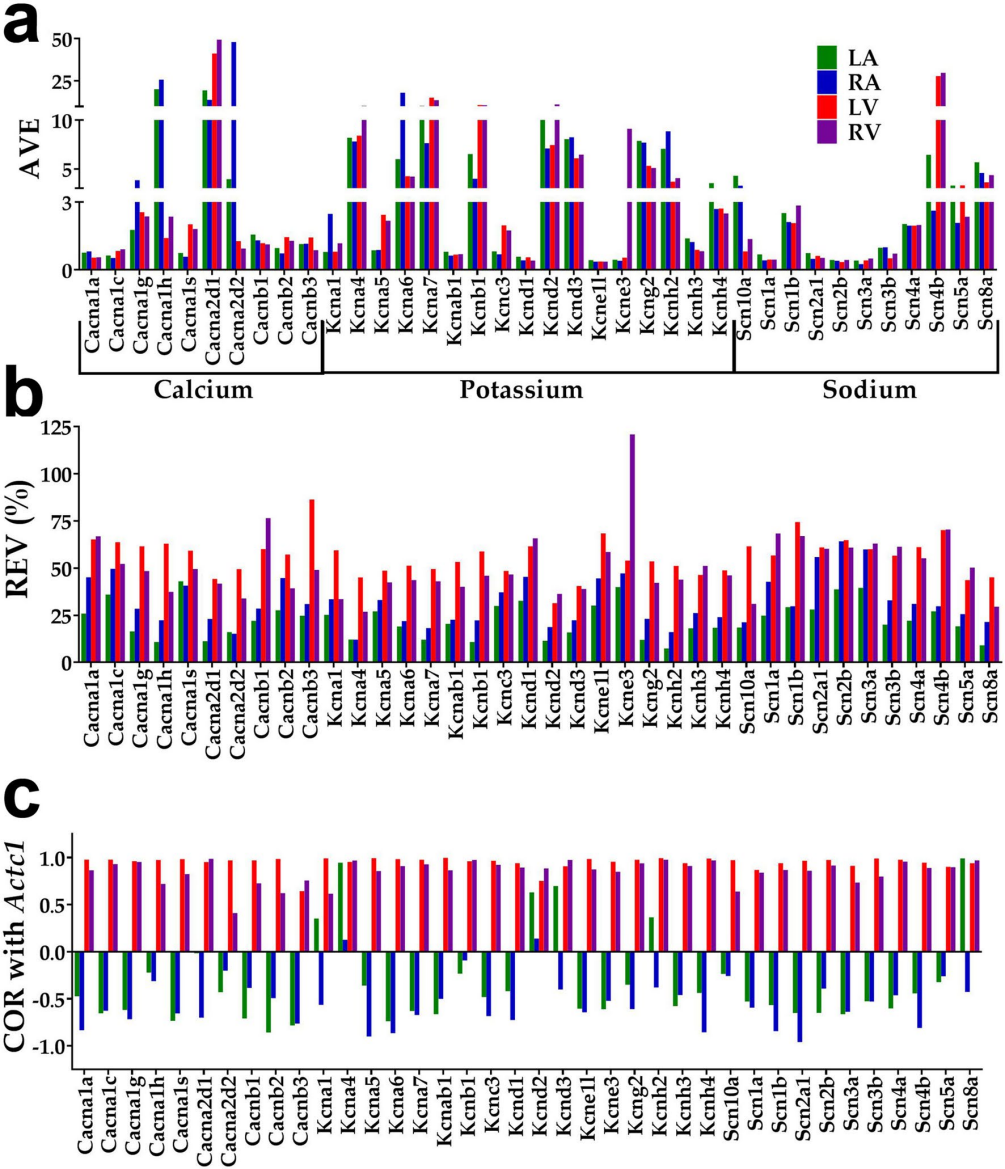
The three independent characteristics of voltage-dependent calcium, potassium and sodium channels in the left atrium (LA), right atrium (RA), left ventricle (LV) and right ventricle (RV). (**a**) Average expression level (AVE); (**b**) (relative) expression variation (REV); (**c**) expression correlation (COR with *Actc1*). AVE unit is the normalized median of all genes quantified in all samples, so AVE = 20 indicates that expression of *Cacna1h* in LA is 20 × larger than that of the median gene in all 16 samples. Expression variation is quantified by the Relative Expression Variation (REV) and expression correlation is measured by COR, the Pearson product-momentum correlation coefficient between the (log_2_) expression levels of the two genes across the biological replicas (see “Methods”).

Figure 1 presents these three features for voltage-dependent calcium, potassium and sodium ion channels. Expression variation was quantified as REV (Relative Expression Variability) (see “Methods”) to correct the expression coefficient of variation among biological replicates for redundant spots probing the same transcript. Owing to its importance for the cardiac muscle contraction and high expression in all chambers, *Actc1* (actin, alpha, cardiac muscle 1) was selected as a reference gene to illustrate the independence of COR with respect to AVE and REV in each chamber. However, coordination with any other gene would also prove the independence of the three features.

Albeit evident just by visual inspection, the independence of the three characteristics for this subset of genes in each chamber was confirmed by the GraphPad Prism v.7.0 (https://www.graphpad.com/scientific-software/prism/) one-way ANOVA test that returned for each chamber and group of two characteristics p-values below 0.00001.

There are several interesting findings about the selected genes reported in Fig. 1. First, the channel genes with the highest average expression levels in all chambers are subunits of the voltage-dependent calcium channels: the main subunit of T-type Ca^2+^ channel *Cacna1h* (20 in LA) and the auxiliary subunits of L-type Ca^2+^ channels *Cacna2d2* (48 in RA), and *Cacna2d1* (41 in LV and 49 in RV). This observation reveals the importance of ion channel density in controlling the heart rhythm^30^. However, as shown in Supplementary Tables S1–S3, out of all quantified 199 ICT genes, the largest AVE were for *Atp5b* (1112 in LA, 795 in RA, 811 in LV, 1082 in RA) and *Kctd14* (1075 in LA, 825 in RA, 834 in LV, 825 in RA).

Second, almost all illustrated genes have smaller REVs in atria than in ventricles, meaning that the expression of these genes are more controlled in atria than in ventricles.

Third, almost all analyzed genes in Fig. 1c are negatively correlated with *Actc1* in atria and positively correlated in ventricles, indicating that the voltage-dependent ion channels are oppositely related to the myocardial contraction^30,31^ in the two types of chambers. Thus, expression of most ion channels increases and decreases together with expression of *Actc1* in ventricles while in atria they manifest opposite tendencies. Owing to its symmetry to the gene permutation, the coordination analysis cannot decide about the expression change of what gene triggers change of the other.

Supplementary Tables S1–S3 present the AVEs and REVs of several types of ion channels in the left atrium (LA), right atrium (RA), left ventricle (LV) and right ventricle (RV).

### Differential expression, control and prominence among the heart chambers

**Figure 2:**
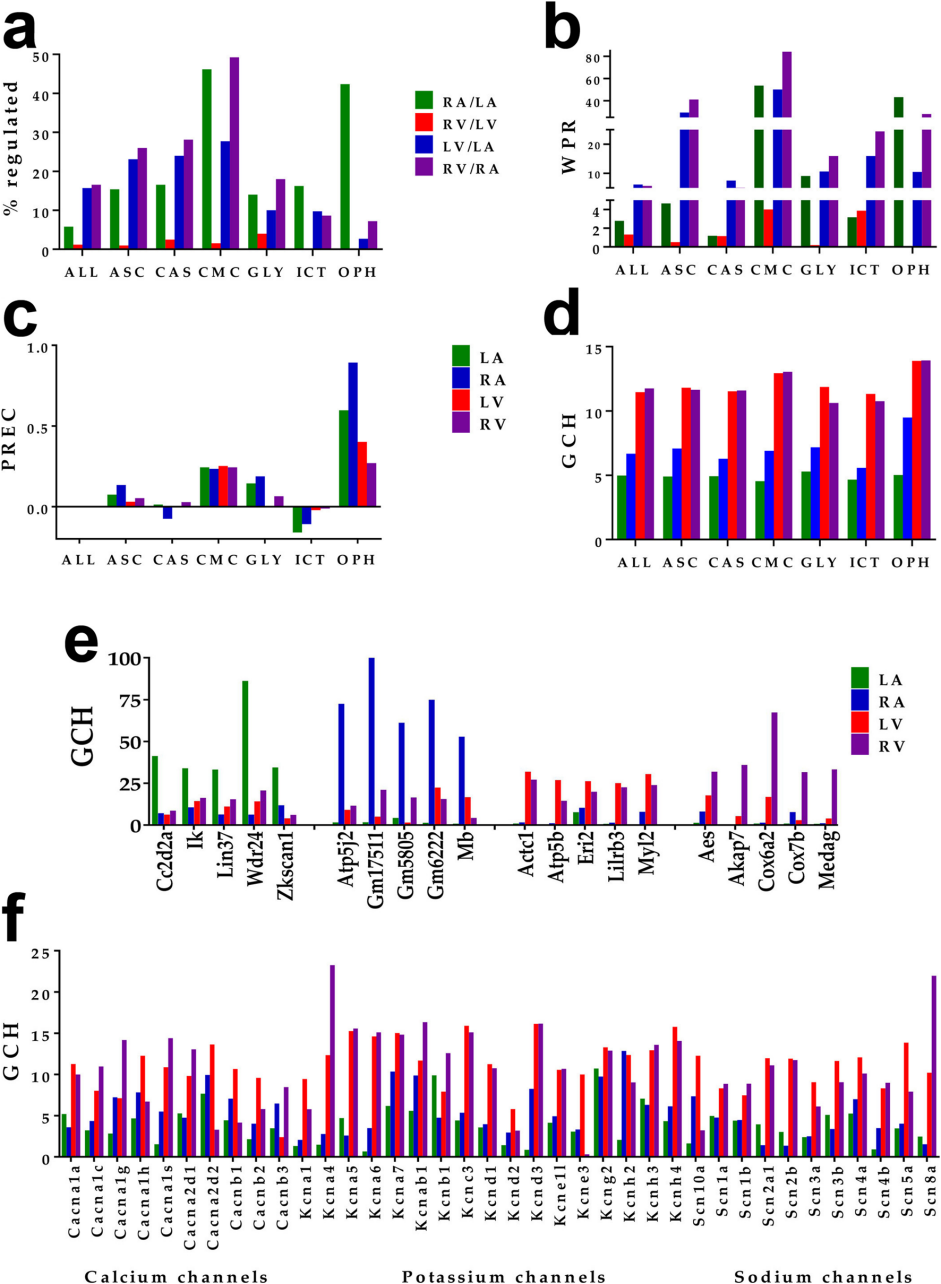
Differential expression, control and prominence of selected functional pathways and groups of genes in LA, RA, LV, RV = left and right atria and ventricles. (**a**) Percentages of regulated genes; (**b**) weighted pathway regulation (WPR); (**c**) pathway relative expression control; (**d**) top 5 genes (highest GCH scores) in each chamber; (**e**) five most prominent (highest GCH) genes in each chamber; (**f**) GCH scores of voltage-dependent calcium, potassium and sodium channels. The denominator in the legend of panels (**a**) and (**b**) indicates the reference chamber, while the numerator indicates the referred one. *ALL* all genes, *ASC* adrenergic signaling in cardiomyocytes, *CAS* calcium signaling, *CMC* cardiac muscle contraction, *GLY* glycolysis/gluconeogenesis, *ICT* ion channels and transporters, *OPH* oxidative phosphorylation. Note that CMC is the most altered, OPH the most controlled and ICT the least controlled pathway.

In Fig. 2, panels (a) and (b) present the percentage of differentially expressed genes and the Weighted Pathway Regulation (WPR^32^) of selected pathways when comparing the two atria, the two ventricles and the ventricle and atrium of the same side. The scores of all quantified genes (ALL) were included for comparison. While significant differences between the atrium and the ventricle from the same side of the heart were expected, of note are the large differences between the two atria and the very small differences between the two ventricles. The high WPR scores for the CMC pathway when comparing the two atria and each atrium to the ventricle of the same side come from the very large expression levels of genes encoding contractile myofilaments. Interestingly, while genes such as: *Actc1, Tnnt2, Tpm1* have very large expression in all chambers, others have significantly larger expression in atria (*Myl4*) or in ventricles (*Myl2, Myl3*). The average expression levels for these genes were: *Actc1* (1685 in LA, 1089 in RA, 1541 in LV, 1310 in RV), *Tnnt2* (1290, 732, 1132, 1118), *Tpm1* (1255, 905, 1256, 1193), *Myl4* (1963, 1127, 55, 46), *Myl2* (108, 408, 1081, 1335) and *Myl3* (314, 412, 1725, 1735).

Figure 2c shows the Pathway Relative Expression Control (PREC) and Supplementary Table S4 the Relative Expression Controls (REC) of the individual ICT genes. The most controlled genes are: *Vdac2* (REC = 4.66 in LA), *Atp5j2* (13.53 in RA, 2.17 in LV), *Atp5b* (2.15 in RV).

The average GCHs of the selected pathways in each heart chamber are presented in Fig. 2d. Figure 2e presents the GCHs of the top five genes in each chamber and Fig. 2f the GCH scores of calcium, potassium and sodium voltage-dependent channels. Note in Fig. 2e that the chambers have distinct hierarchies and in Fig. 2f that no voltage-dependent ion channel makes the top 5 genes in any chamber. However, although none of the top five genes in LV are among the top five in RV, they perform similarly in both ventricles.

In previous works, e.g.^28^, we proved the transcriptomic integration of hetero-cellular tissues by which each cell phenotype adjusts its transcriptome to optimize the intercellular communication and tissue physiology. However, in this study, we were particularly interested in genes expressed exclusively by the cardiomyocytes such as those encoding subunits of cardiac-specific voltage-dependent ICTs. Supplementary Table S5 lists the expression ratios between right and left ventricle (RV/LV) and between right and left atria (RA/LA) for 12 genes encoding subunits of cardiac-specific ICTs.

### Chamber specificity of gene expression within functional pathways

**Figure 3:**
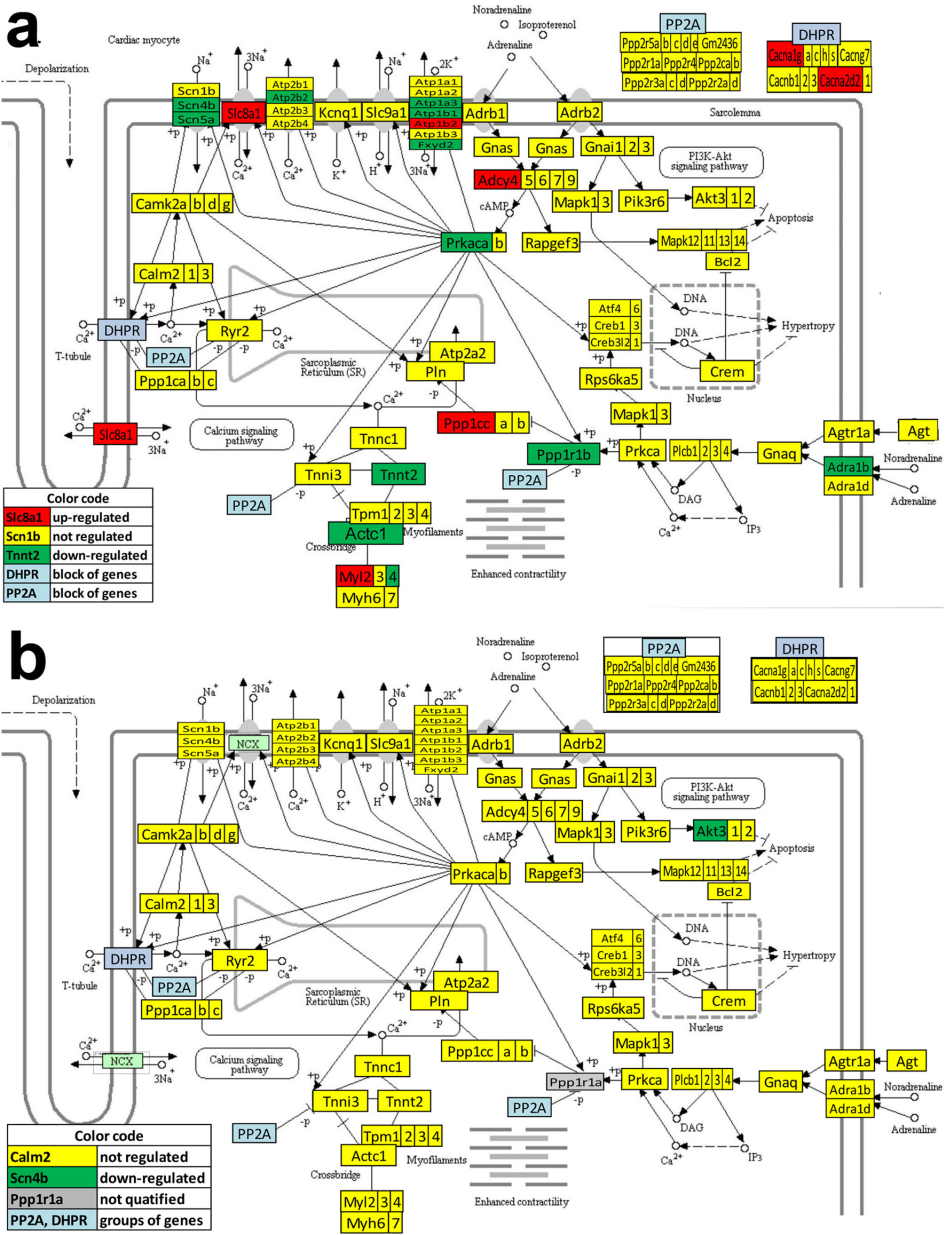
KEGG map ( modified from https://www.genome.jp/dbget-bin/www_bget?path:mmu04261) of the differential expression of adrenergic signaling in cardiomyocytes (ASC) genes from: (**a**) the right atrium with respect to the left atrium, (**b**) the right ventricle with respect to the left ventricle. Only *Akt3* was found with significantly lower expression in the right ventricle with respect to the left one. Red/green/yellow background of the gene symbol indicates up-/down-/not regulated. Genes with significant differences: actin alpha cardiac muscle 1 (*Actc1*), adenylate cyclase 4 (*Adcy4*), adrenergic receptor alpha 1b (*Adra1b*), angiotensin II receptor type 1b (*Agtr1b*), thymoma viral proto-oncogene 3 ( *Akt3*), Ca^++^ transporting plasma membrane 2 (*Atp2b2*), Na^+^/K^+^ transporting ATPases (*Atp1a3, Atp1b1, Atp1b2*), calcium channels (*Cacna1g, Cacna2d2*), FXYD domain-containing ion transport regulator 2 (*Fxyd2*), light myosins (*Myl2, Myl4*), protein phosphatase 1 regulatory (inhibitory) subunit 1B (*Ppp1r1b*), protein kinase, cAMP dependent, catalytic, alpha (*Prkaca*), sodium channels (*Scn4b*, *Scn5a*), solute carrier family 8 (sodium/calcium exchanger) member 1 (*Slc8a1*), troponin T2 cardiac (*Tnnt2*).

**Figure 4:**
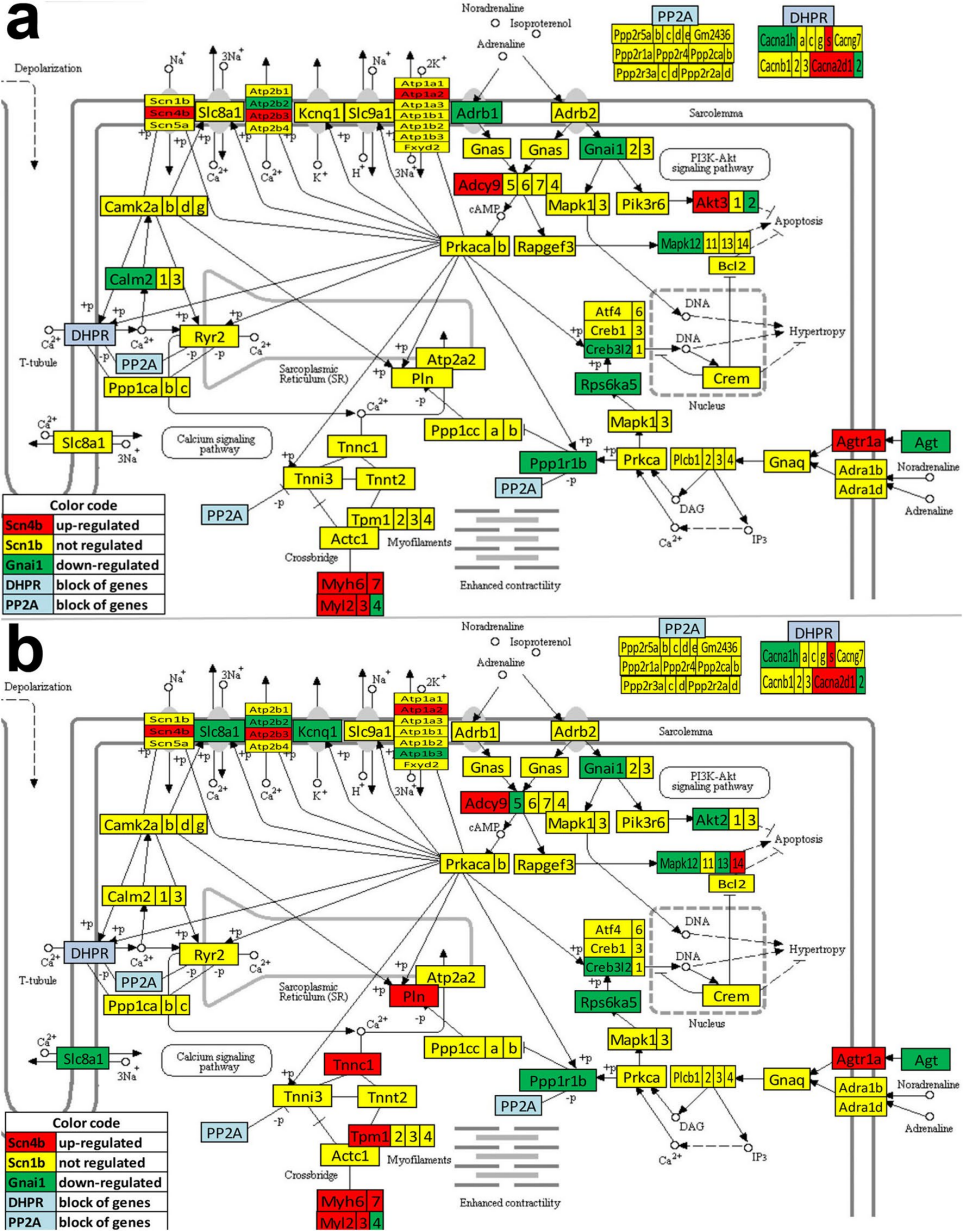
KEGG map ( modified from https://www.genome.jp/dbget-bin/www_bget?path:mmu04261) of the differential expression of adrenergic signaling in cardiomyocytes (ASC) genes from: (**a**) the left ventricle vs left atrium and (**b**) the right ventricle vs right atrium. Red/green/yellow background of the gene symbol indicates up-/down-/not regulated. Genes with significant differences: adenylate cyclases (*Adcy5, Adcy9*), adrenergic receptor beta 1 (*Adrb1*), angiotensinogen (*Agt*), angiotensin II receptor type 1a (*Agtr1a*), thymoma viral proto-oncogenes (*Akt2, Akt3*), Ca^++^ transporting ATPases (*Atp2b2, Atp2b3*), Na^+^/K^+^ transporting ATPases (*Atp1a2*, *Atp1b3*), calcium channels (*Cacna1h, Cacna1s, Cacna2d1, Cacna2d2*), calmodulin 2 (*Calm2*), cAMP responsive element binding protein 3-like 2 (*Creb3l2*), potassium voltage-gated channel subfamily Q member 1 (*Kcnq1*), mitogen-activated protein kinases (*Mapk12, Mapk13, Mapk14*), heavy (*Myh6, Myh*) and light myosins (*Myl2, Myl3, Myl4*), phospholamban (*Pln*), protein phosphatase 1 regulatory (inhibitory) subunit 1B (*Ppp1r1b*), ribosomal protein S6 kinase polypeptide 5 (*Rps6ka5*), sodium channels (*Scn4b*, *Scn5a*), solute carrier family 8 (sodium/calcium exchanger) member 1 (*Slc8a1*), troponin T1, skeletal, slow (*Tnnt1*) and tropomyosin 1 alpha (*Tpm1*).

**Figure 5:**
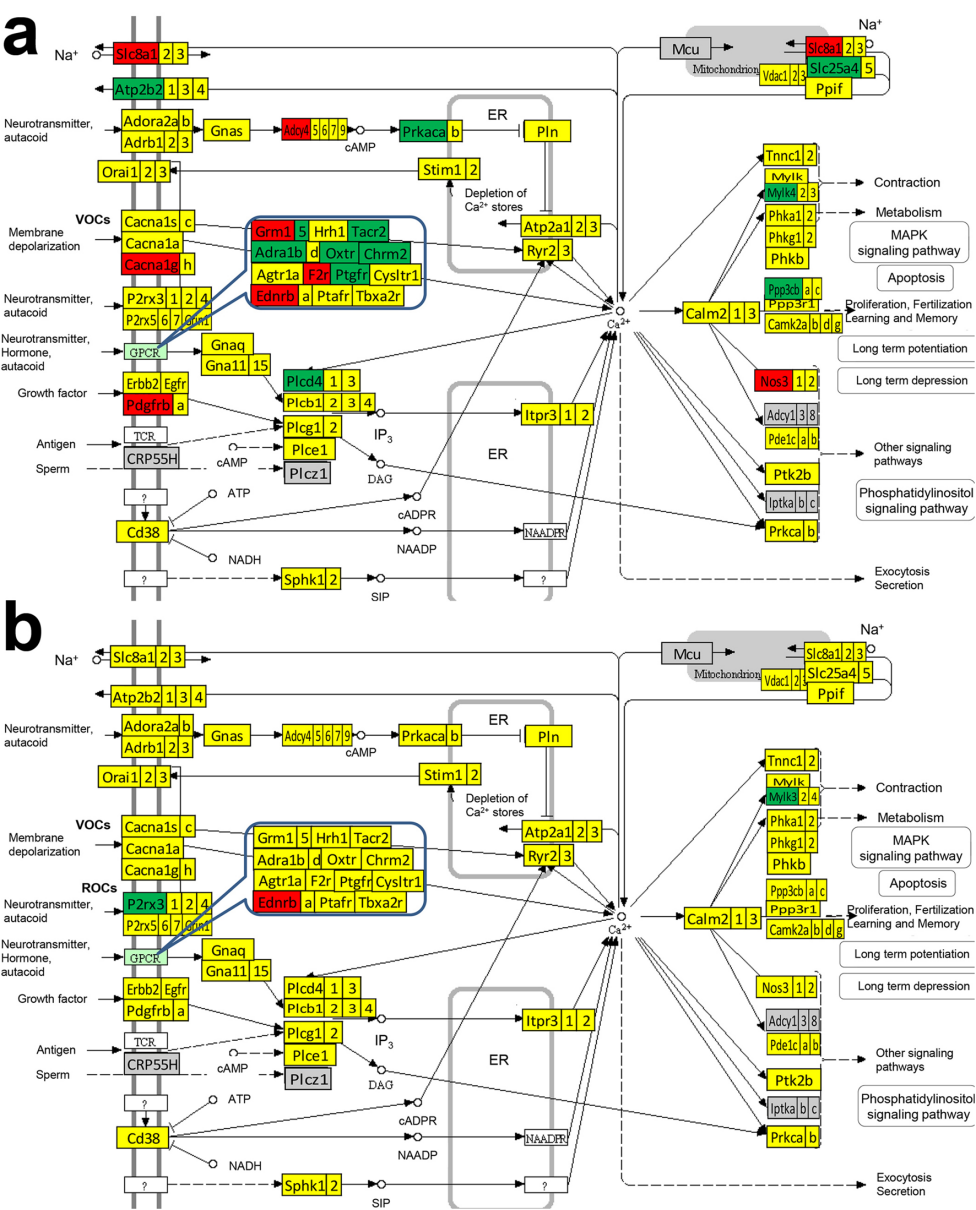
KEGG map ( modified from https://www.genome.jp/dbget-bin/www_bget?mmu04020) of the differential expression of calcium signaling (CAS) genes in: (**a**) the right atrium with respect to the left atrium, (**b**) right ventricle vs left ventricle. Red/green/yellow background of the gene symbol indicates up-/down-/not regulated. Not quantified genes are on grey background. Genes with significant differences: *Adcy4*, adrenergic receptors (*Adra1b*), *Atp2b2*, *Cacna1g,* cholinergic receptor muscarinic 2 cardiac (*Chrm2*), endothelin receptor type B (*Ednrb*), coagulation factor II (thrombin) receptor (*F2r*), metabotropic glutamate receptors (*Grm1, Grm5*), myosin light chain kinase (*Mylk4*), nitric oxide synthase (*Nos3*), oxytocin receptor (*Oxtr*), purinergic receptor P2X ligand-gated ion channel 3 (*P2rx3*), platelet derived growth factor receptor (*Pdgfrb*), phospholipase (*Plcd4*), protein phosphatase 3, catalytic subunit, beta isoform (*Ppp3cb*), protein kinase cAMP dependent catalytic alpha (*Prkac*a), prostaglandin F receptor (*Ptgfr*), solute carriers (*Slc25a4*, *Slc8a1*), tachykinin receptor 2 (*Tacr2*).

**Figure 6:**
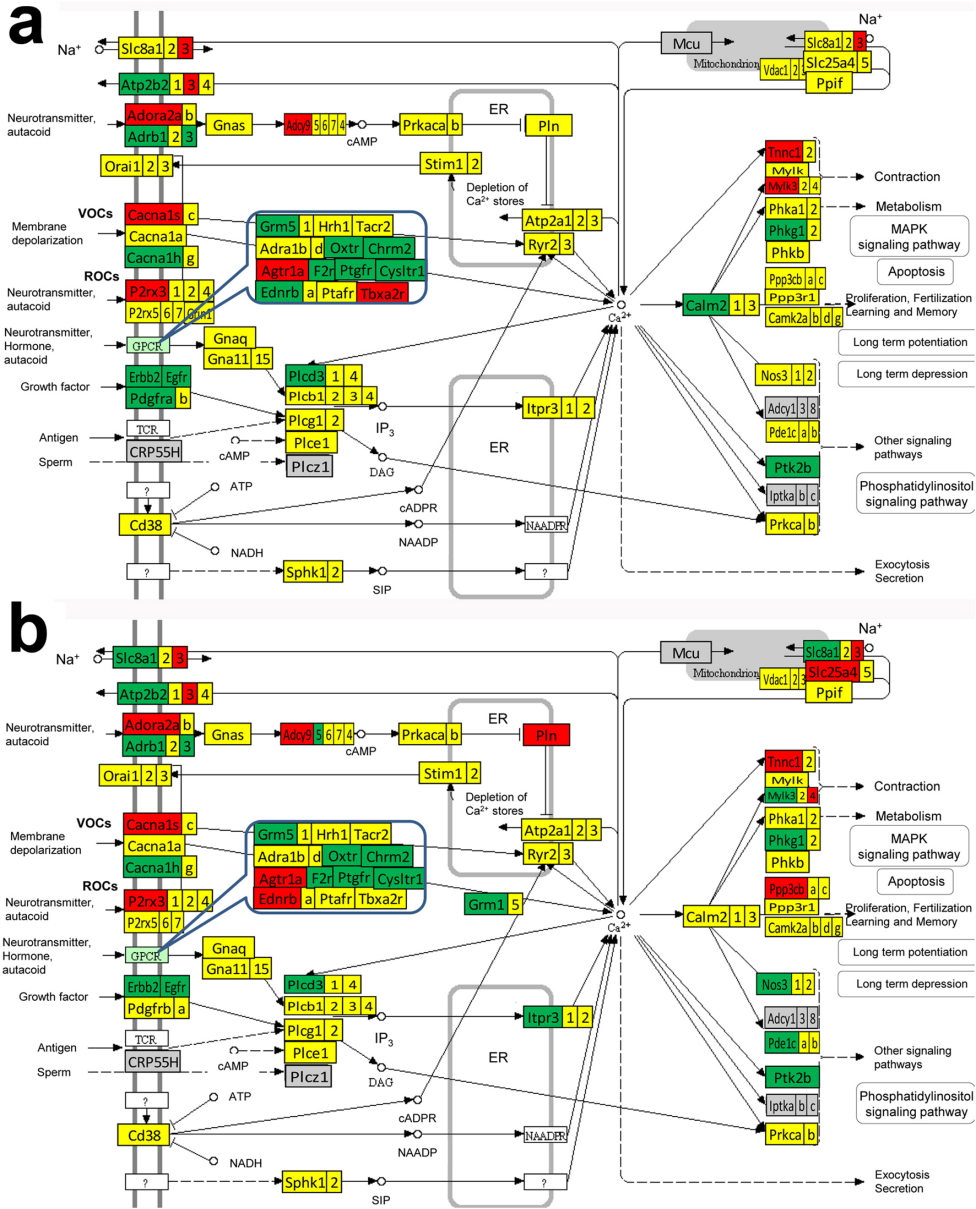
KEGG map ( modified from https://www.genome.jp/dbget-bin/www_bget?mmu04020) of the differential expression of calcium signaling (CAS) genes in: **a.** left ventricle vs left atrium, **b.** right ventricle vs right atrium. Red/green/yellow background of gene symbol indicates up-/down-/not regulated. Red/green/yellow background of the gene symbol indicates up-/down-/not regulated. Not quantified genes are on grey background. Genes with significant differences: *Adcy5, Adcy9*, adenosine A2a receptor (*Adora2a*), adrenergic receptors (*Adrb1, Adrb3*), *Agtr1a*, *Atp2b2, Atp2b3*, *Cacna1h, Cacna1s*, *Calm2*, *Chrm2*, cysteinyl leukotriene receptor 1 (*Cysltr1*), *Ednrb*, epidermal growth factor receptor (*Egfr*), v-erb-b2 erythroblastic leukemia viral oncogene homolog 2 (*Erbb2*), *F2r*, *Grm5*, inositol 1,4,5-triphosphate receptor 3 (*Itpr3*), myosin light chain kinases (*Mylk3, Mylk4*), *Nos3*, *Oxtr*, *P2rx3*, phosphodiesterase 1C (*Pde1c*), *Pdgfra,* phosphorylase kinase gamma 1 (*Phkg1*), *Plcd3,* phospholamban (*Pln*), *Ppp3cb*, *Ptgfr*, PTK2 protein tyrosine kinase 2 beta (*Ptk2b*), *Slc8a1, Slc8a3*, thromboxane A2 receptor (*Tbxa2r*) and troponin C cardiac/slow skeletal (*Tnnc1*).

Figures 3 and 4 present the differentially expressed genes within the ASC pathway between the two atria, between the two ventricles and between the atrium and the ventricle from the same side. Figures 5 and 6 present the differentially expressed genes within the CAS pathway between the two atria, between the two ventricles and between the atrium and the ventricle from the same side. Supplementary Figs. S1–S3 present the differentially expressed genes within CMC, GLY and OPH pathways between the two atria and between the atrium and the ventricle of each side of the heart.

While the differences between the atrium and the ventricle from the same side were expected, of note in all these figures are the substantial gene expression differences between the two atria and the much smaller differences between the two ventricles.

### Expression correlation and synchrony of ion channels and transporters

**Figure 7:**
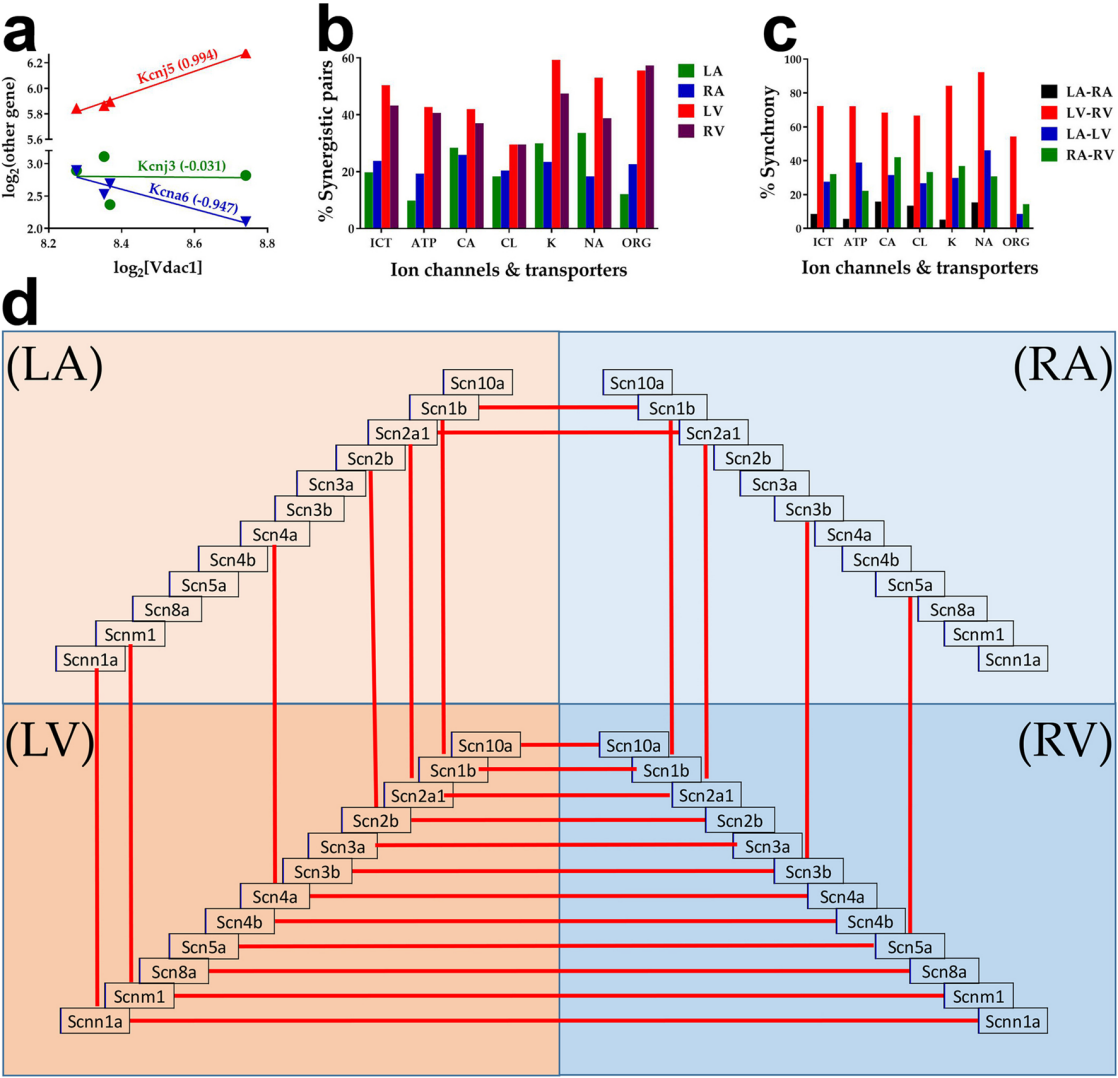
Correlation of the expressions of genes encoding ion channels and transporters (ICT). (**a**) Examples of synergistically (*Kcnj5* = potassium inwardly-rectifying channel, subfamily J, member 5), antagonistically (*Kcna6* = potassium voltage-gated channel, shaker-related, subfamily, member 6) and independently (*Kcnj3* = potassium inwardly-rectifying channel, subfamily J, member 3) expressed genes with *Vdac1* (voltage-dependent anion channel 1) in the left atrium. (**b**) Percentage of synergistically expressed ion channel and transporter gene pairs within each chamber. (**c**) Synchronous expressions of ICT genes between two chambers. (**d**) Synchronous expressions of sodium channels in paired heart chambers are indicated by red lines linking the gene symbols. The correlation was computed for each ICT gene with each other ICT gene within the same chamber. *ICT* percentage out of all 17,020 pairs that can be formed with the quantified ICT genes, *ATP* transporting ATPases (1378 pairs), *ORG* transporters through organelle (mitochondria, lysosomes) membranes (903 pairs), *CA* calcium channels (153 pairs), *CL* chloride channels (91 pairs), *NA* sodium channels (91 pairs), *K* potassium channels (1596 pairs). Note the significantly larger percentages of synergistically expressed pairs of ion channels and transporters in the ventricles.

Figure 7 presents examples of significantly (p-val < 0.05) synergistically, antagonistically and independently expressed ICT genes (a), the percentages of significant synergistic expressions of the ICT genes with each-other in each chamber (b) and of the same ICT genes between two adjacent chambers (c). Panel d shows the sodium channels that were synergistically expressed in two adjacent chambers. The in-phase fluctuation of gene transcripts in two adjacent chambers indicates expression synchrony of that gene in those chambers. We found that expression synchrony of ICTs is significantly higher between the two ventricles than between the two atria.

**Figure 8:**
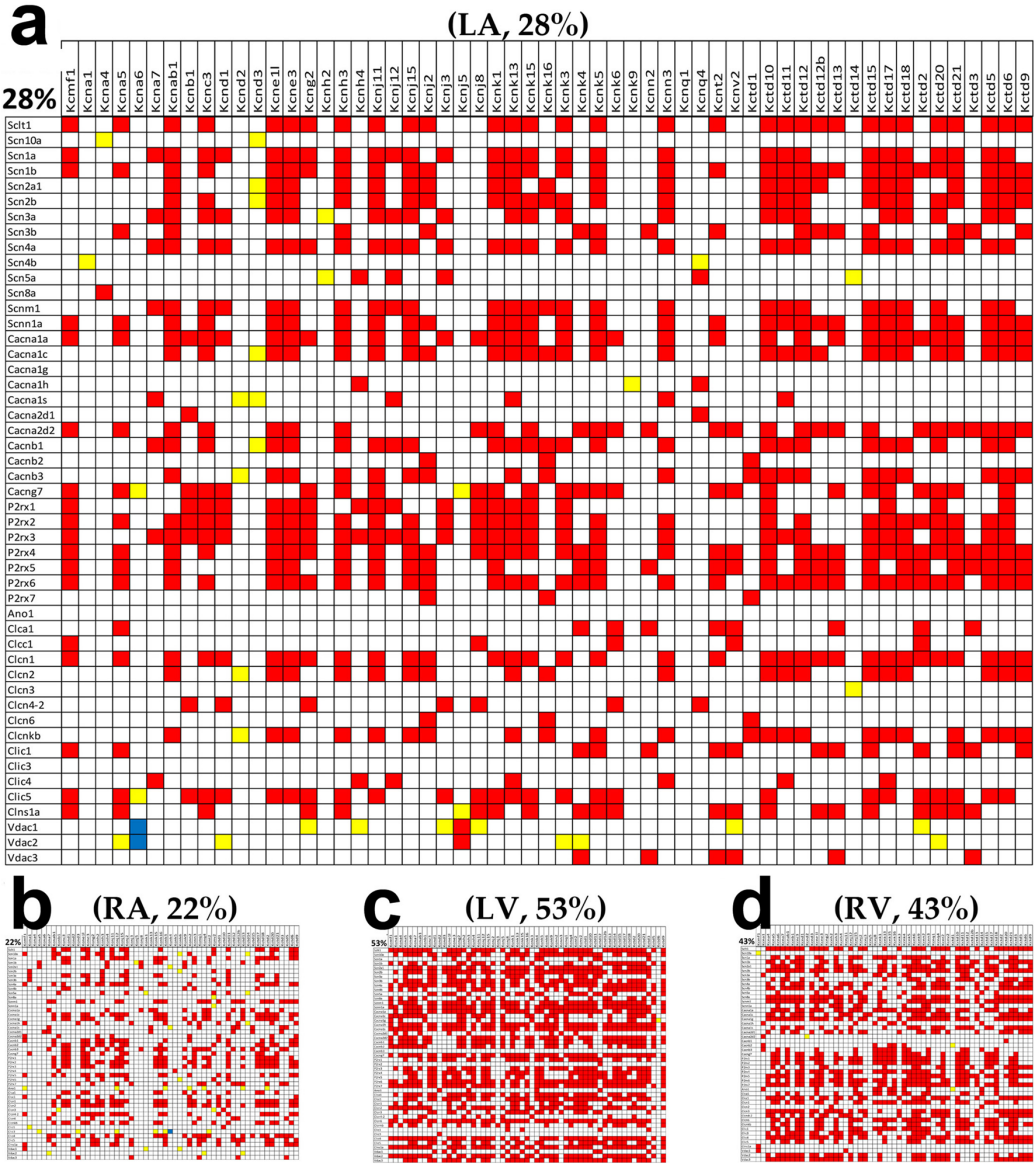
Significant (p < 0.05) expression correlation and independence of the 57 potassium channel genes with the 49 sodium, calcium and chloride channel genes in (**a**) left atrium, (**b**) right atrium, (**c**) left ventricle, (**d**) right ventricle. Red/blue/yellow square indicates that the potassium channel gene labeling the column and the sodium/calcium/chloride channel labeling the intersected row are significantly synergistically/antagonistically/ independently expressed. Numbers above the graphs represent the percentages of synergistic pairs within the gene selection in the indicated chamber.

Figure 8 shows the significantly (p-val < 0.05) synergistically, antagonistically and independently expressed genes out of the 2,793 pairs formed by the 57 potassium channel genes with the 49 sodium, calcium and chloride channel genes, in the four chambers of the male mouse heart. While there are less than 1% significant antagonistic and independent pairs, the percentages of the synergistically expressed pairs are very high in all chambers, although with remarkable differences among them: 28% in the LA, 22% in the RA, 53% in the LV and 43% in the RV.

### Expression coordination of Ankyrin B with its potential binding partners

Ankyrin-B (encoded by *Ank2*) is an essential cytoskeletal component that performs integral (transmembrane) protein anchoring, containing four primary domains: a membrane-binding, a spectrin-binding, a death, and a C-terminal, each with binding partners^26,33^. Pearson correlation coefficients between the expression levels of *Ank2* and those of its potential binding partners were computed for each chamber. The analysis aimed to validate the partners whose transcripts abundances oscillate in phase with that of *Ank2* among biological replicas.

**Figure 9:**
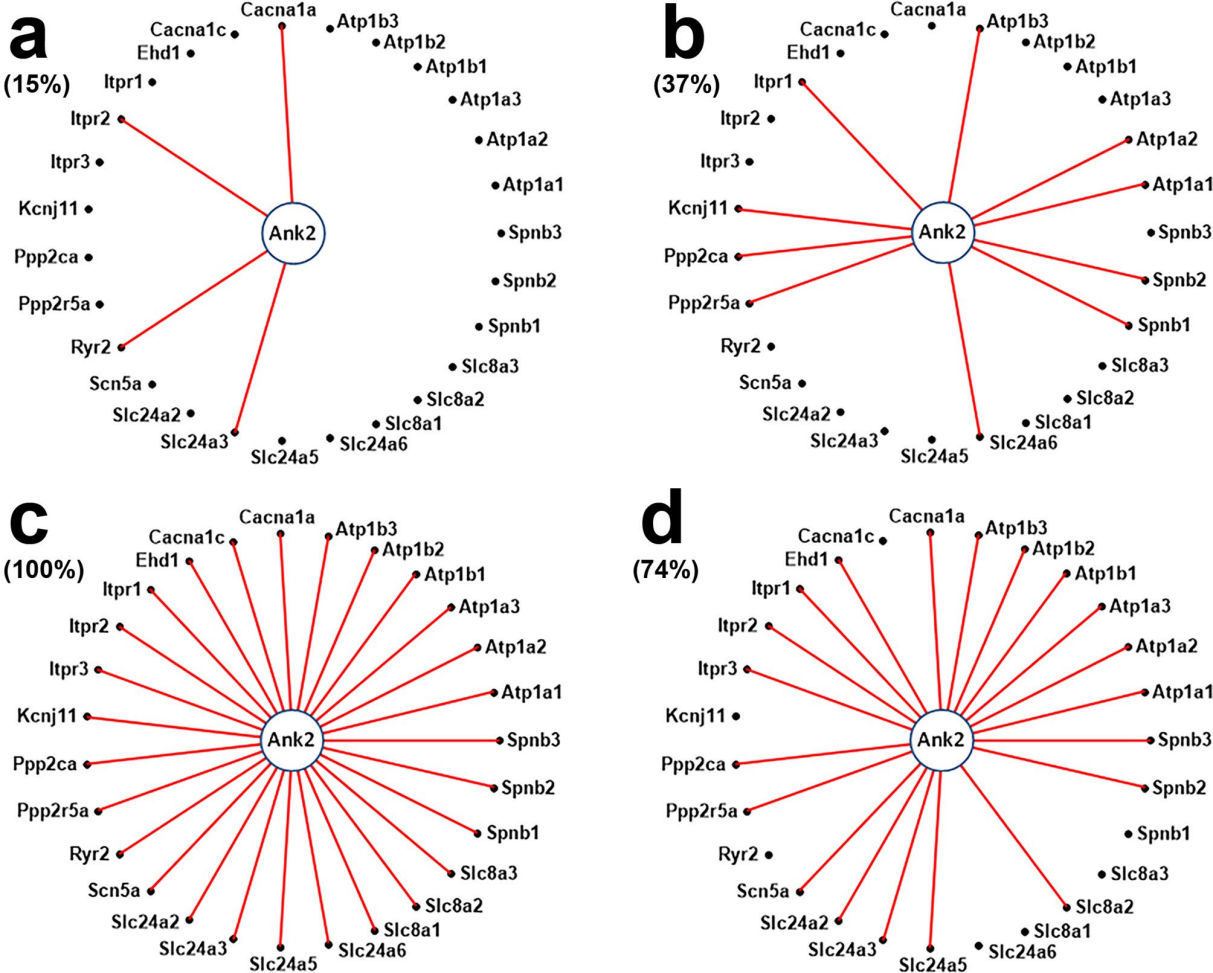
Expression coordination of *Ank2* with its known binding partners in: (**a**) left atrium, (**b**) right atrium, (**c**) left ventricle, (**d**) right ventricle. Genes: *Atp1a1/2/3* (ATPase, Na^+^/K^+^ transporting, alpha 1/2/3 polypeptide), *Atp1b1/2/3* (ATPase, Na^+^/K^+^ transporting, beta 1/2/3 polypeptide), *Cacna1a* (calcium channel, voltage-dependent, P/Q type, alpha 1A subunit), *Cacna1c* (calcium channel, voltage-dependent, L type, alpha 1C subunit), *Ehd1* (EH-domain containing 1), *Itpr1/2/3* (inositol 1,4,5-trisphosphate receptor 1/2/3), *Kcnj11* (potassium inwardly rectifying channel, subfamily J, member 11), *Ppp2ca* (protein phosphatase 2 (formerly 2A), catalytic subunit, alpha isoform), (protein phosphatase 2, regulatory subunit B (B56), alpha isoform), *Ryr2* (ryanodine receptor 2, cardiac), *Scn5a* (sodium channel, voltage-gated, type V, alpha), *Slc24a2/3/5/6* (solute carrier family 24 (sodium/potassium/ calcium exchanger), member 2/3/5/6), *Slc8a1/2/3* (solute carrier family 8 (sodium/calcium exchanger), member 1/2/3), *Spnb1/2/3* (spectrin beta 1/2/3).

Figure 9 and Supplementary Figs. S4–S6 present the significant expression coordination of *Ank2, Ctnnb1, Hspa5* and *Mapk1* with *Akt2* potential binding partners in the four heart chambers. All these four genes were identified in a recent paper^27^ as key players in regulating the cardiac rhythm. While no statistically significant antagonism or independence was found in any of the four genes’ networks, the percentages of significant synergisms within this selection of genes were substantially larger in ventricles than in atria. Thus, *Akt2* was synergistically expressed with 15% of its partners in LA, 37% in RA, 100% in LV and 74% in RV. *Ctnnb1* (19% in LA, 0% in RA, 93% in LV and 74% in RV), *HSpa5* (19% in LA, 26% in RA, 85% in LV), and 70% in RV), and *Mapk1* (11% in LA), 22% in RA), 96% in LV and 70% in RV).exhibited qualitatively similar networks.

## Discussion

A PubMed search for “mouse cardiac gene expression” returned 22,271 results on 10/13/2020, separate profiling of heart chambers in the same animal/pool of animals revealing chamber-specific patterns of gene expression from perinatal^34^ to adult stages^14^. However, unique for our study is the GFP perspective^28^ that increases by several orders of magnitude the transcriptomic information collectible from a gene expression experiment. Thus, besides the traditional AVE, GFP assigns to each gene the independent characteristics REV and COR with each other gene from the same chamber (or with the same gene in an adjacent chamber). Our procedure increases the accuracy of detecting significant differential expression by replacing the uniform absolute fold-change cut-off with CUT determined separately for each gene in each two-chamber comparison function of biological variability and technical noise. Moreover, WPR weights the contribution of each pathway’s gene to the pathway alteration by its reference expression level, expression ratio and p-value of the differential expression. WPR is a much more comprehensive alternative to the traditional percentage of differentially expressed genes that is limited to the genes satisfying arbitrarily introduced cut-offs and considers all the regulated genes as equal contributors. In addition, we determined also the chamber differences in gene expression variability and inter-coordination. REV and COR analyses were further combined to establish the gene hierarchy and identify the GMR of each chamber.

A very interesting observation in Fig. 1c is that almost all ICT genes are negatively correlated with *Actc1* in both atria but positively correlated in both ventricles that may partially explain the differential effects of ion channels activity on atria and ventricles. For instance, owing that atrial myocytes lack transverse tubules and have more abundant corbular sarcoplasmic reticulum, local Ca^2+^-signaling and by consequence the contraction forces differ between the two types of chambers, e.g.^35^. If proved by further functional analysis, the opposite correlations of ICT genes with the one encoding the major protein responsible for contraction of thin filament in cardiac sarcomere may have important applications in the differential treatment of atrial and ventricular cardiomyopathies.

In previous publications^29,36,37^, we have shown that expression correlations with one gene predicts with > 80% accuracy the regulation of its coordinately expressed partners when the expression of the target gene is significantly altered. Therefore, according to our results from Fig. 1c, blocking expression of certain ion channels should decrease the expression of *Actc1* in ventricles (positive correlation) but increase it in atria (negative correlation). These results provide an alternative explanation for the effectiveness of calcium blockers^38^ in the treatment of hypertension and why lowering the blood pressure prevents atrial fibrillation^39^, Our hypothesis does not contradict but complements the widely accepted view that the anti-hypertensive effects of calcium blockers and regulation of the atrial rhythm result from changes in the heart electrophysiology and hemodynamics. We speculate that the increase of the atrial contraction strength acts synergistically with the reduction of the ventricular hypertrophy following an anti-hypertensive treatment to lower the atrial difficulties in filling with blood the ventricle from the same side. This hypothesis goes along with the reported differences in Ca^2+^ cycling and its role in controlling the myocyte contraction in atria and ventricles^40^.

Although we have analyzed numerous pathways and coordination of all gene pairs that can be formed, owing to the limited space, this report presents only the pathways and the individual genes and gene pairs considered by many authors among the most important for the heart physiology. However, the publically available expression data (GSE45339, GSE45348) and our “CORRELATION” software^41^ allows any interested researcher to determine the inter-correlation of any group of genes. Unfortunately, the huge volume of transcriptomic data and the lack of adequate resources at the time prevented us to validate the genomic results through functional analyses. Nonetheless, as detailed in the cited literature, most of our transcriptomic results are in agreement with functional observations of other authors.

Atrial and ventricular myocardia are heterocellular tissues^42^ where, besides the cardiomyocytes (the majority of cells), fibroblasts, nerve endings, immune system cells, endothelial and smooth muscle cells, stem cell niches and other supporting cells are also present. Though we did our best to dissect and profile as homogeneous as possible small regions from the muscular walls of each heart chamber, the cardiomyocyte RNA was inevitably mixed with unknown amounts of RNA from non-cardiomyocyte cells. Nevertheless, as we proved in previous publications, e.g.^28,43,44^ by profiling mouse cortical astrocytes and oligodendrocytes cultured alone and co-cultured in insert systems, the organization of each cell type transcriptome is significantly modeled by the heterogeneous cellular environment. Hence, gene expression analysis of mono-cellular cardiomyocyte cultures gives a different picture than that of the cardiomyocytes embedded in the myocardium, although it is hard to estimate how much different. Unfortunately, even single-cell RNA-sequencing^45^, applied to heterogeneous tissues is affected by technical errors such as sampling biases, changes in expression during enzyme digestion or pre-processing heart perfusion^46^. Hence, a good approach is to focus analysis on cardiomyocyte-specific genes. Therefore, we provided in Supplementary Table S5 the expression ratios for several cardiomyocyte ICT genes.

By examining the expression levels of the ATPases presented in Supplementary Table S1, we noticed that on average the expression of H^+^ mitochondrial transporters is with 33% larger in ventricles, while the lysosomal transporters are with 41% more expressed in atria. These observations may explain the serious consequences of ischemic cardiomyopathy^47^ (that reduces the proton pumping in mitochondrial respiration) on the left ventricle^48^ and the severe bradyarrhythmia of the Fabry disease (lysosomal storage disorder linked to atrial dysfunction^49^).

Some ion channels (listed in Supplementary Tables S2, S3, S5) also feature differential atrio-ventricular expression. Thus, *Kcnj5*, encoding the inward-rectifier K^+^ channel K_ir_3.4, a type of G protein-gated ion channel contributing the acetylcholine-activated current, is preferentially expressed in the atria, accounting for the specificity of vagal control of heart rate and conduction velocity. Point mutations of this channel (a.k.a. *GIRK4* or *KATP1*) may result in a peculiar type of long QT syndrome (LQT-13), as well as in familial hyperaldosteronism type III^50^. *Kcnq1* encoding Kv7.1 or KvLQT1, the ion channel contributing the slow component of the cardiac delayed rectifier K^+^ current *I*_*Ks*_, is also preferentially expressed in atria. This ion channel, activated by β-adrenergic stimulation, provides a “repolarization reserve” that is very useful during physical exercise^51^; it is also the cause of the most widespread type of long QT syndrome, LQT-1^52,53^. Although evidenced in isolated human ventricular cardiomyocytes^54^, patch-clamp experiments on isolated mouse ventricular myocytes failed to identify a distinct *I*_Ks_^53,55^. The levels of expression of certain chloride channels (Supplementary Table S3) were higher in atrial myocardium (*Clcc1, Clic3, Clcn42*—possibly involved in stretch activation and cell volume regulation), while others were preferentially expressed in ventricles (*Clcn1, Clcn3* and *Clic5*).

While the high expression levels of mitochondrial H^+^ transporters in all chambers is understandable given the energy needs for the sustained muscular effort, those of *Kctd14* (potassium channel tetramerisation domain containing 14) came as a surprise. Not only the observed high expressions are against the negligible values reported by NCBI sequencing data for the adult mouse heart (https://www.ncbi.nlm.nih.gov/gene/233529), but for now there is no PubMed report about any role of *Kctd14* in the heart physiology. We believe that the implication of *Kctd14* in the heart contraction (presumably by regulating the tetramerisation of the potassium channels) deserves future research.

The expression levels of ion channels of the transient receptor potential (TRP) family, shown in Supplementary Table S3, are even more interesting. Thus, while most TRP genes show equal expression in the four heart chambers, *Trpm4* and *Trpm7* are predominantly expressed at the atrial level. *Trpm4* was shown to be present at sino-atrial node level, where it influences pacemaking^56^; its properties, such as selective permeability for monovalent cations, activation by calcium and reversal potential between + 10 and + 30 mV, are superposable to those of the “sustained inward” Na^+^ and K^+^ current (*I*_st_) identified in sino-atrial pacemaker cells^57^. *Trpm7* performs an astounding diversity of functions, including intracellular Mg^2+^ homeostasis and transport of Zn^2+^, Cu^2+^, Fe^2+^^58,59^. Its presence in ventricular^60^ and atrial^61^ cardiomyocytes was demonstrated by electrophysiology and mRNA expression. Several studies reported increased TRPM7 basal current levels in human atrial cardiomyocytes dissociated from patients with coronary heart disease or arterial hypertension^62^, speculating about its role in atrial fibrillation, fibrosis^63^ and electrical remodelling^64^. Other important TRP channel with preferential ventricular over atrial expression is *Trpc3*, important in both store-operated and receptor-operated Ca^2+^ entry^54^. *Trpc3* overexpression induces myocardial hypertrophy via calcineurin/NFAT in transgenic mice^65^, while left ventricular pressure overload by transverse aortic constriction or angiotensin-II infusion upregulated TRPC non-selective cation current levels in cardiomyocytes^66^.

Interestingly, as evidenced in Fig. 1b, the median REVs in atria (21 in LA, 29 in RA) are substantially smaller than in ventricles (57 in LV and 48 in RV). Since the REV is inversely related to the strength of the cell homeostatic mechanisms to keep transcript abundances within narrow intervals, these results indicate a much stricter control^67^ of the expression of voltage-dependent ion channels in atria than in ventricles.

We found several statistically significant transcriptomic differences among the four chambers. The differences were the largest between the atrium and ventricle of same side, substantially large between the right and left atria but almost negligible between the right and left ventricles, both for the entire transcriptome and for the selected pathways. Our results for male heart were qualitatively similar to what was previously reported for adult female C57Bl/6 mice^14^, although our analysis was on male heart (of the same mouse strain) and went far beyond the expression levels. The differences between the right and the left atria (RA/LA) were larger for the cardiac muscle contraction (CMC) and oxidative phosphorylation (OPH) pathways.

We hypothesize that the transcriptomic differences among the chambers were accumulated during the evolution of the cardiovascular system, with the largest (between atria and ventricles) indicating first stage of heart evolution and the smallest (between the two ventricles) the last one. Indeed, the initial separation of the contracting vessel into one atrium and one ventricle, present since the beginning of the chordate phylum, was followed by the separation into right and left atria that started with amphibians. Separation into right and left ventricle started with the archosaurs (crocodilians, dinosaurs and pterosaurs)^68^. Nevertheless, the right and the left ventricles have different embryogenetic origins. Thus, LV and part of the atria arise from the early heart tube developed from the first heart field, an area of anterior splanchnic mesoderm formed early during gastrulation. In turn, RV, the outflow tract and the main parts of atrial tissue arise from the second heart field that coalesces with the heart tube at its arterial and venous edges, being derived from the pharingeal mesoderm and dorsal mesocardium^54^.

Differential gene expression proved that the anterior intestinal portal endoderm is a heart organizer, capable to induce cardiac identity into non-cardiac mesoderm even if heterotopically transplanted, and patterning cardiac tissue to express ventricular and suppress atrial region identifiers^54,69^. Moreover, single-cell RNA-seq was used to understand the earliest steps of cardiovascular lineage differentiation, analyzing MesP1-positive cardiovascular progenitor cells and different cardiac progenitor populations corresponding to the first and second heart fields at embryonic days E6.5 and E7.25 in mice^70^.

The intricate cardiomyocyte differentiation involves a complex interplay of signalling pathways and gene regulatory networks^6^, families of activating transcription factors^8^, transcription enhancers^70^ and epigenetic mechanisms^7,71^. The analysis of the calcium signaling pathway from the WPR perspective (Fig. 2b) revealed substantial differences between the atrium and ventricle from the same side, as expected given the important but distinct roles of Ca^2+^ in the physiology of the two types of chambers^72–74^. However, we found practically no difference for this pathway between the two atria and between the two ventricles, a possible indication of Ca^2+^-homeostasis similarity in the left and right heart. Also, practically no difference was found for the β-adrenergic receptor genes between the two ventricles. Differences were rather found between the ventricle and the atrium of the same side for associated membrane proteins involved in ion transport as effectors, subunits of the Na^+^/K^+^ pumps, Na^+^/Ca^2+^ exchangers (NCX), L-type Ca^2+^ channels and voltage-dependent Na^+^ channel subunits. We found also significantly larger expression in ventricles for the angiotensin II receptor type Ia (*Agtr1a*) whose abnormal expression was linked to hypertension and cardiac hypertrophy^75^. These results are relevant for the roles of adrenergic signaling and internal calcium dynamics in the “fight-or-flight” response to sympathetic nerve stimulation, essential for the positive inotropic response to stress-generating situations^76^.

We did not perform the pathway enrichment analysis because all pathways built by specialized software (KEGG, PathVisio, GenMAPP, Ingenuity Pathway Analysis, GO-Elite, DAVID, Cytoscape etc.) are artificially universal, unique and rigid that we consider far from reality. Such constructed pathways are universal because their internal organization is the same regardless of race/strain, sex, age, tissue, medical history, (micro and macro) environmental conditions etc. They are unique because they provide only one wiring of the genes, although even two chemical elements (H & C) can combine to form a wide variety of hydrocarbons. Finally, they are rigid because the gene interconnections do not change during maturation/ageing, progression of a disease or response to external stimuli. By contrast, numerous published reports have shown that the pathways: (1) depend on race^41^/strain^77^, tissue^22^, sex^11^, age^67,77^, medical history^47^, environmental conditions^67^ etc. Although KEGG software was used to select the genes and illustrate the regulation of certain pathways, we have shown that genes may be interlinked in various ways (Fig. 8), and the genomic fabric of some functional pathways remodel in disease^47^ and recovers in response to a well conducted treatment^32,78^.

An interesting finding in Fig. 2c is that PREC has the largest values for the OPH pathway in all chambers, indicating that the energy metabolism is the most controlled pathway, presumably to limit alteration of the ATP level^79^. In contrast, ICT is the least controlled group of genes, most likely for faster adaptation to the needed changes in heart rhythm, electrical and contractile properties during physical activity^80^. However, the low expression control of ICT genes is compensated by the high percentages of their synergistic pairing (particularly in LV, Fig. 7b) and expression synchrony (particularly between the two ventricles, Fig. 7c,d). We assume that the high interventricular expression synchrony of the sodium channels and their poor interatrial synchrony may be the evolutionary results of much earlier separation of the two atria.

Remarkable is also the high expression synergism of *Ank2* with ICT group (Fig. 9), justifying the important role of Ankyrin-B in cardiac physiology as observed through functional studies of other authors^33^. Particularly important is its significant synergism with *Kcnj11* (a.k.a. *Kir6.2*)^81^.

An interesting finding that can explain the atrio-ventricular gene expression differences is the preferential expression of certain homebox genes such as *Dkk3* (dickkopf WNT signaling pathway inhibitor 3) and, *Pdlim3* (PDZ and LIM domain 3) and *Hmgb1* (high mobility group box 1) at atrial level, and *Pdlim1* (elfin), *Fhl2* (four and a half LIM domains 2) and *Hmgb3* (high mobility group box 3) at ventricular level^14^. The large transcriptomic differences found between the two atria and the much smaller ones between the two ventricles can be linked to the unequal cell proliferation rates along and across the primitive heart tube and changes in spatial orientations^82^. Nevertheless, the differences between the atria and the ventricles go beyond the expression level. One may observe in Fig. 7 that the expression synchrony of the sodium channels between the adjacent chambers is practically perfect between the ventricles and very poor between the atria.

Figure 2e shows that, beyond their orchestrated contractions, each heart chamber has a specific gene hierarchy, indicating distinct transcriptomic organizations. As expected, among the most prominent genes in LV are: *Actc1* and *Myl2* (myosin light polypeptide 2 regulatory cardiac, slow). Interestingly, within the top 5 genes in all chambers, there are 4 mitochondrion genes: ATP synthase, H^+^ transporting, mitochondrial complexes (*Atp5b, Atp5j2*) and cytochrome c oxidase subunits (*Cox6a2, Cox7b*). There are also 3 predicted genes: *Gm17511, Gm5805, Gm6222*, all in the right atrium that deserve functional analyses to better understand their roles in the heart physiology. Of note is also the high GCH in the left atrium of the subunit (*Lin37*) of the dimerization partner, RB-like, E2F and multi-vulval class B (DREAM) complex that control the cell cycle^83^. Our results explain why each chamber may develop a distinct pathology that needs a chamber-specific treatment. However, the top five genes in LV have similar GCHs in RV, indicating that a myocardium-targeted treatment for LV problems may be also efficient for RV.

## Methods

### Tissues

Four adult (18–19 months, 32–34 g) male C57Bl/6j mice, purchased from Charles River Laboratories International, Inc. (Wilmington, MA, USA) were used to profile separately the myocardia transcriptomes of each heart chamber. The study was limited to only males because the female transcriptome depends on the estrogen level^13^ and changes during the estrous cycle^12^. Therefore, a complete study on females not only would quadruplicate the work to profile the heart chambers of animals synchronized in each of the cycle phases, but would be affected by the non uniform estrogen level.

The animals were housed in rooms with controlled temperature (22 ± 2 °C) and humidity (55 ± 10%), continuous air flow and 12 h light/12 h dark cycle (6 am–6 pm), were provided with normal rodent diet and water ad libitum, and monitored daily by trained veterinary personnel at Albert Einstein’s Accredited Research Animal Care and Use Facility (https://www.einstein.yu.edu/research/shared-facilities/cores/52/animal-housing-and-studies). The mice were decapitated under light isoflurane anesthesia and the hearts were isolated and perfused with saline to wash out all remaining blood. All experiments were carried out according to the approved #20100205 protocol (PI DA Iacobas) by the (Einstein) Institutional Animal Care and Use Committee (IACUC) for prevention of disease, daily observation and surveillance for assessment of animal health, and the methods of animal handling, restraint, anesthesia, and analgesia.

### Microarray

1 mm sized as homogeneous as possible pieces were isolated from the lateral walls of LA, RA, LV and RV, not close to the SA or AV node, or the bundle of His or origin of its branches in the interventricular septum. Total RNA was immediately extracted in separate vials with RNAEasy Minikit (Qiagen, Germantown, MD, USA), following manufacturer's instructions. RNA concentration was determined before and after reverse transcription in the presence of Cy3/Cy5 dUTP with a WU-83060-00 Thermo Scientific NanoDrop ND-1000 and its quality with a 2100 Bioanalyzer (Agilent, DE). 825 ng of differently (Cy3/Cy5) labeled biological replicas of the same chamber were hybridized 17 h at 65 °C with GPL10333 Agilent-026655 Whole Mouse Genome Microarray 4 × 44 K v2. The chips were washed and scanned with an Agilent G2539A dual laser scanner at 5 μm resolution in 20-bit scan mode and primary analysis performed with (Agilent) Feature Extraction 11.6 software.

Agilent microarrays and Illumina NextSeq 500 (equally available to us) provide similar expression ratios^84^. However, in addition to being considerably cheaper, microarrays were preferred because their open protocol allowed optimization to significantly reduce the technical noise. More importantly, microarray raw data are of reasonable size to allow the extensive COR analysis with the available computer resources.

### Data analysis

We have used our standard protocol^28^ for data filtration and normalization. Any spot with corrupted pixels or with foreground fluorescence less than twice the background in any of the 16 samples was removed from the analysis. As justified in a recent paper^28^, profiling four biological replicas provides for the expression of each gene three independent measures: level, variability and correlation with each other gene from the same chamber or with the same gene in adjacent chamber. The three features are as independent and complementary as are the impressions on the same movie of a blind person and a deaf one.

#### Average expression level

Agilent mouse 4 × 44 k two-color microarrays can hybridize up to 30,175 distinct transcripts, out of which 22,657 are probed by single spots. However, other genes are probed by more spots. The most redundantly probed (by 13 spots) are: *Abcc5, Cpne4, Csf1, Esr1, Mapk1, Oprm1, P2rx3, Socs2.* Therefore, we average the expression level of each gene first on biological replicas for the same spot and then over all spots probing redundantly the same gene.

1 $$ AVE_{i}^{(chamber)} = \frac{1}{{R_{i} }}\sum\limits_{k = 1}^{{R_{i} }} {\mu_{i,k}^{(chamber)} } = \frac{1}{{R_{i} }}\sum\limits_{k = 1}^{{R_{i} }} {\underbrace {{\left( {\frac{1}{4}\sum\limits_{j = 1}^{4} {a_{i,k,j}^{(chamber)} } } \right)}}_{{\mu_{i}^{(chamber)} }}} , $$

where *chamber* = *LA*, *RA*, *LV*, *RV*; R_*i*_ = number of spots probing redundantly gene *i*; $a_{i,j,k}^{(chamber)}$ = expression level of gene “*i*” probed by spot “*k*” on biological replica “*j*” in “*chamber*”

### Expression variability and control

Because of the transcript probing redundancy, we used the mid-interval chi-square estimate of the coefficient of variation (CV) of the normalized expression of each gene in the profiled chamber, adjusted for multiple spots, termed REV^28^.

2 $$ \begin{gathered} \forall chamber = LA,RA,LV,RV \hfill \\ REV_{i}^{(chamber)} = \underbrace {{\frac{1}{2}\left( {\sqrt {\frac{{r_{i} }}{{\chi^{2} \left( {r_{i} ;0.975} \right)}}} + \sqrt {\frac{{r_{i} }}{{\chi^{2} \left( {r_{i} ;0.025} \right)}}} } \right)}}_{{\text{redundancy correction coefficient}}}\underbrace {{\sqrt {\frac{1}{{R_{i} }}\sum\limits_{k = 1}^{{R_{i} }} {\left( {\frac{{s_{ik}^{(chamber)} }}{{\mu_{ik}^{(chamber)} }}} \right)^{2} } } }}_{{{\text{pooled CV}}_{i}^{(chamber)} }} \times 100\% \hfill \\ \end{gathered} $$

$s_{ik}^{(chamber)}$ = standard deviation of the expression level of gene *i* probed by spot *k* in “chamber”; *r*_*i*_ = 4*R*_*i*_ – 1 = number of degrees of freedom

For mouse Agilent microarrays, REV corrects the CV by a factor ranging from 1.566 (for R = 1) to 1.05 (R = 13).

REV was further used to compute the REC of individual genes and the PREC of selected pathways:

3 $$ REC_{i}^{(chamber)} = \frac{{\left. {\left\langle {REV_{{}}^{(chamber)} } \right\rangle } \right|_{ALL} }}{{REV_{i}^{(chamber)} }} - 1\quad ,\quad PREC_{\Gamma }^{(chamber)} = \frac{{\left. {\left\langle {REV_{{}}^{(chamber)} } \right\rangle } \right|_{ALL} }}{{\left. {\left\langle {REV_{{}}^{(chamber)} } \right\rangle } \right|_{\Gamma } }} - 1 $$

where $\left. {\left\langle {REV_{{}}^{(chamber)} } \right\rangle } \right|_{\Gamma /ALL}$ ≡ median of REV over pathway Γ/entire transcriptome

**H**igher positive *REC* values indicate genes whose expression level is strongly controlled by the cellular homeostatic mechanisms to limit their fluctuations within narrow intervals, critical for the cell survival, phenotypic expression or/and integration in the multicellular structure of the myocardium. By contrast, lower negative *REC*s are associated with less controlled genes that be used as cell adaptors to the slight fluctuations of the environmental conditions, as seen in the biological replicas. Similarly, high *PREC*s are associated with critically important pathways to preserve the phenotype against slight fluctuations of the environment and low *PREC*s with adapting pathways. One may observe that $REC_{i}^{(chamber)} = 0\quad \& \quad PREC_{ALL}^{(chamber)} = 0$ set the baseline for genes and pathways comparisons according to their *PREC* score.

#### Differential expression

A gene was considered as significantly differentially expressed between the compared tissues if the absolute expression ratio |*x*| (*x* negative for down-regulation) exceeded the cut-off *CUT* computed for that gene and the p-value of the heteroscedastic *t-*test of means’ equality was p < 0.05:

4 $$ \begin{gathered} \forall A,B = RA,\;LA,\;RV,\;LV \hfill \\ \left| {x_{i}^{(A \to B)} } \right| > CUT_{i}^{(A \to B)} = 1 + \sqrt {2\left( {\left( {REV_{i}^{(A)} } \right)^{2} + \left( {REV_{i}^{(B)} } \right)^{2} } \right)} \quad ,\quad where: \hfill \\ x_{i}^{(A \to B)} = \left\{ {\begin{array}{*{20}l} {\sum\limits_{k = 1}^{{R_{i} }} {\mu_{ik}^{(B)} } /\sum\limits_{k = 1}^{{R_{i} }} {\mu_{ik}^{(A)} } } \hfill & {if} \hfill & {\sum\limits_{k = 1}^{{R_{i} }} {\mu_{ik}^{(B)} } \ge \sum\limits_{k = 1}^{{R_{i} }} {\mu_{ik}^{(A)} } } \hfill \\ { - \sum\limits_{k = 1}^{{R_{i} }} {\mu_{ik}^{(A)} } /\sum\limits_{k = 1}^{{R_{i} }} {\mu_{ik}^{(B)} } } \hfill & {if} \hfill & {\sum\limits_{k = 1}^{{R_{i} }} {\mu_{ik}^{(B)} } < \sum\limits_{k = 1}^{{R_{i} }} {\mu_{ik}^{(A)} } } \hfill \\ \end{array} } \right. \hfill \\ \end{gathered} $$

As defined, *CUT* takes into account the combined effect of the technical noise of all redundantly probing spots of that gene and the biological variabilities of the transcript abundances in the compared samples.

In addition to the popular percentage of significantly regulated genes according to the above criterion (that replace the traditional uniform absolute fold-change cut-off of 1.5 × or 2.0x), we quantify the alteration of the functional pathways by the Weighted Pathway Regulation (WPR) redefined from^32^:

5 $$ \begin{gathered} WPR_{\Gamma }^{(A \to B)} = \overline{{\left( {wpr_{i}^{(A \to B)} } \right)_{i \in \Gamma } }} \quad ,\quad where: \hfill \\ wpr_{i}^{(A \to B)} = \left\{ {\begin{array}{*{20}l} {\mu_{i}^{(A)} \left( {\left| {x_{i}^{(A \to B)} } \right| - CUT_{i}^{(A \to B)} } \right)\left( {1 - p_{i}^{(A \to B)} } \right)} \hfill & {if} \hfill & {\left| {x_{i}^{(A \to B)} } \right| > CUT_{i}^{(A \to B)} } \hfill \\ 0 \hfill & {if} \hfill & {\left| {x_{i}^{(A \to B)} } \right| \le CUT_{i}^{(A \to B)} } \hfill \\ \end{array} } \right. \hfill \\ p_{i}^{(A \to B)} {\text{ = p - val of the heteroscedastic t - test of }}\mu_{i}^{(B)} = \mu_{i}^{(A)} \hfill \\ \end{gathered} $$

#### Expression coordination

Expression variability within biological replicas allowed us to compute the Pearson product-moment correlation coefficient between the (log_2_) expressions of each gene *i* across biological replicas with each other gene *j* in the same chamber or with the same gene in adjacent chambers. We used the correlation analysis to identify significantly (p-val < 0.05) synergistically, antagonistically and independently expressed genes in the same chamber as well synchronous and asynchronous expression of the same gene in adjacent chambers. The statistical significance of the correlation coefficient was determined with the publically available software https://www.youtube.com/watch?v=Kc3M5x7125A (two-tail *t*-test) for degrees of freedom df = 4 (biological replicas)*R (number of spots probing redundantly each of the correlated transcripts) − 2. If each gene was probed by one spot, then df = 4 − 2 = 2, for two spots df = 8 − 2 = 6, for 3 spots df = 12 − 2 = 10. For unequal number of spots, we used for each biological replica the average expression level of all spots probing that gene.

Correlation results were further used to determine the differences of the gene networks among the four heart chambers. We used such analysis previously to determine the remodeling of gene networks in hearts of mice subjected to chronic constant or intermittent hypoxia^67^, with Chagas cardiomyopathy^32^, or with knocked-out expression of Cx43, the main gap junction protein in cardiomyocytes^36^.

#### Gene commanding height and gene master regulators

The GMR was defined as the gene whose tightly controlled expression by the cellular homeostatic mechanisms is a major modulator of most functional pathways by expression coordination with their genes^29^. Using the above defined *REC*, heart GMRs are the genes with the highest Gene Commanding Height (GCH) in each chamber, computed as:

6 $$ \begin{gathered} GCH_{i}^{(chamber)} = \left( {REC_{i}^{(chamber)} + 1} \right) \times \exp \left( {4\overline{{\left. {\rho_{ij}^{2} } \right|_{\forall j \ne i}^{(chamber)} }} } \right) \hfill \\ \, \hfill \\ \end{gathered} $$

We used GCH to identify the genes whose significat altered expression may selectively kill the cancer cells in a heterogeneous tumor^85^.

## Supplementary Information

Supplementary Information
