# Supplementary material for: Transcriptomic uniqueness and commonality of the ion channels and transporters in the four heart chambers
Source: Sci Rep. 2021 Feb 2;11:2743. doi: 10.1038/s41598-021-82383-1 (PMC7854717; doi:10.1038/s41598-021-82383-1)

## Transcriptomic uniqueness and commonality of the ion channels and transporters in the four heart chambers

Sanda Iacobas^1^, Bogdan Amuzescu^2^ and Dumitru A Iacobas^3,4^*

**Supplementary Table 1: Average expression level (AVE) and Relative Expression Variability (REV) of ATPase and ATPsynthase ion transporters in the left (L) and right (R) atrium (A) and ventricle (V).** Grey background indicates the genes with the highest AVE and the highest RV in each chamber.

**Supplementary Table 2: Average expression level (AVE) and Relative Expression Variability (REV) of other potassium channels (not included in Fig.1) in the left (L) and right (R) atrium (A) and ventricle (V).** Grey background indicates the genes with the highest AVE and the highest RV in each chamber.

**Supplementary Table 3: Average expression level (AVE) and Relative Expression Variability (REV) of additional types of ion channels in the left (L) and right (R) atrium (A) and ventricle (V).** Grey background indicates the genes with the highest AVE and the highest RV in each chamber.

**Supplementary Table 4: Relative Expression Control (REC) in the left (L) and right (R) atrium (A) and ventricle (V).**

**Supplementary Table 5: Expression ratios of 12 genes encoding subunits of cardiac ion channels and transporters.** Red/green background of the ratio indicates significant differential expression between the compared chambers (negative if the expression level in denominator chamber is larger (see Methods).

**Supplementary Figure 1: KEGG map (**modified from https://www.kegg.jp/kegg-bin/show_pathway?mmu04260**) of the** **differentially expressed genes within the Cardiac Muscle Contraction (CMC) pathway in (a)** the right atrium with respect to the left atrium, **(b)** left ventricle vs left atrium, **(c)** right ventricle vs right atrium. Red/green/yellow background of gene symbol indicates up-/down-/not regulated**.** No significant difference was found between the expressions of CMC genes in the two ventricles.

**
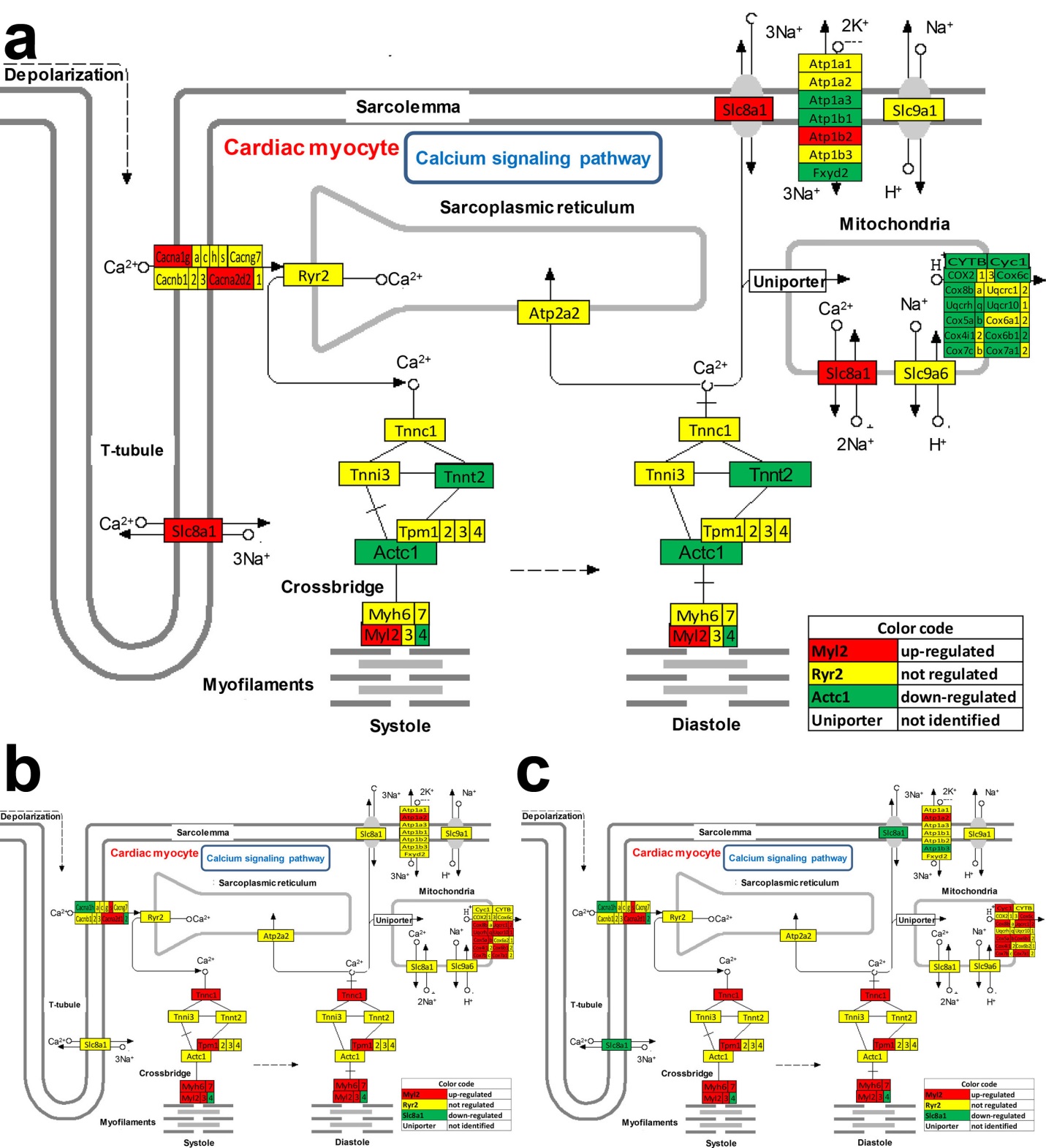
**

**Supplementary Figure 2:** KEGG map (modified from https://www.genome.jp/dbget-bin/www_bget?path:mmu00010) of the differential expression of glycolysis/gluconeogenesis (GLY) genes in: **(a)** the right atrium with respect to the left atrium, **(b)** right ventricle vs left ventricle, **(c)** left ventricle vs left atrium, **(d)** right ventricle vs right atrium. Red/green/yellow background of gene symbol indicates up-/down-/not regulated. **Genes with significant differences:** acyl-CoA synthetase short-chain family member 1 (*Acss1*), alcohol dehydrogenase 1 (*Adh1*), aldehyde dehydrogenases (*Aldh1b1, Aldh3a1*), aldolases (*Aldoa, Aldob, Aldoc*), 2,3-bisphosphoglycerate mutase (*Bpgm*), enolase 3 beta muscle (*Eno3*), fructose bisphosphatase 2 (*Fbp2*), glyceraldehyde-3-phosphate dehydrogenase (*Gapdh*), phosphoenolpyruvate carboxykinase 2 (*Pck2*), phosphoglycerate mutase 1 (*Pgam1*) and pyruvate kinase muscle (*Pkm2*).

**
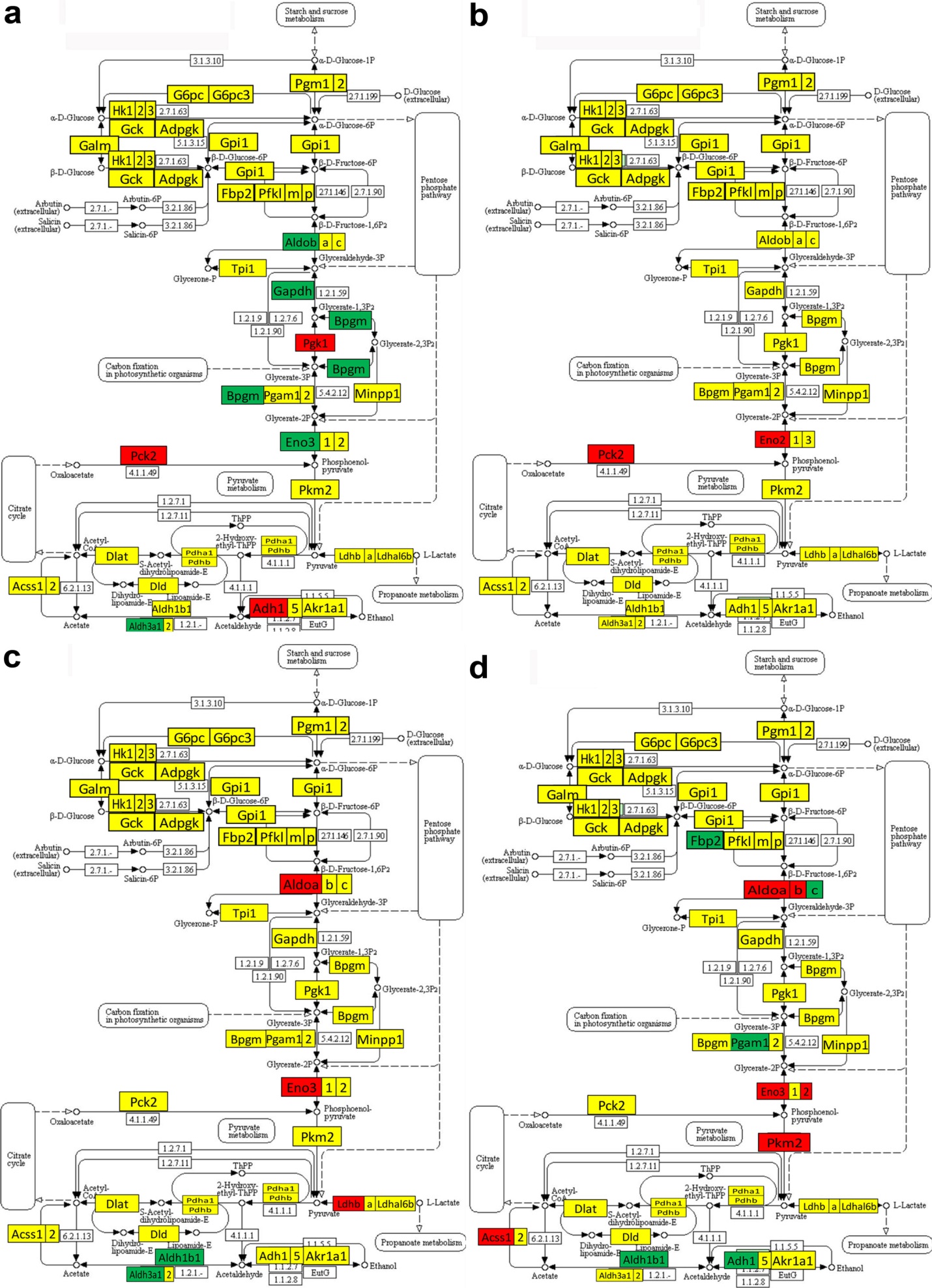
**

**Supplementary Figure 3:** KEGG map (modified from <https://www.kegg.jp/kegg-bin/show_pathway?mmu00190>) of the differential expression of oxidative phosphorylation (OPH) genes in: **(A)** the right atrium with respect to the left atrium, **(B)** left ventricle vs left atrium and **(C)** right ventricle vs right atrium. No significant difference was found between the expressions of OPH genes in the two ventricles.

**
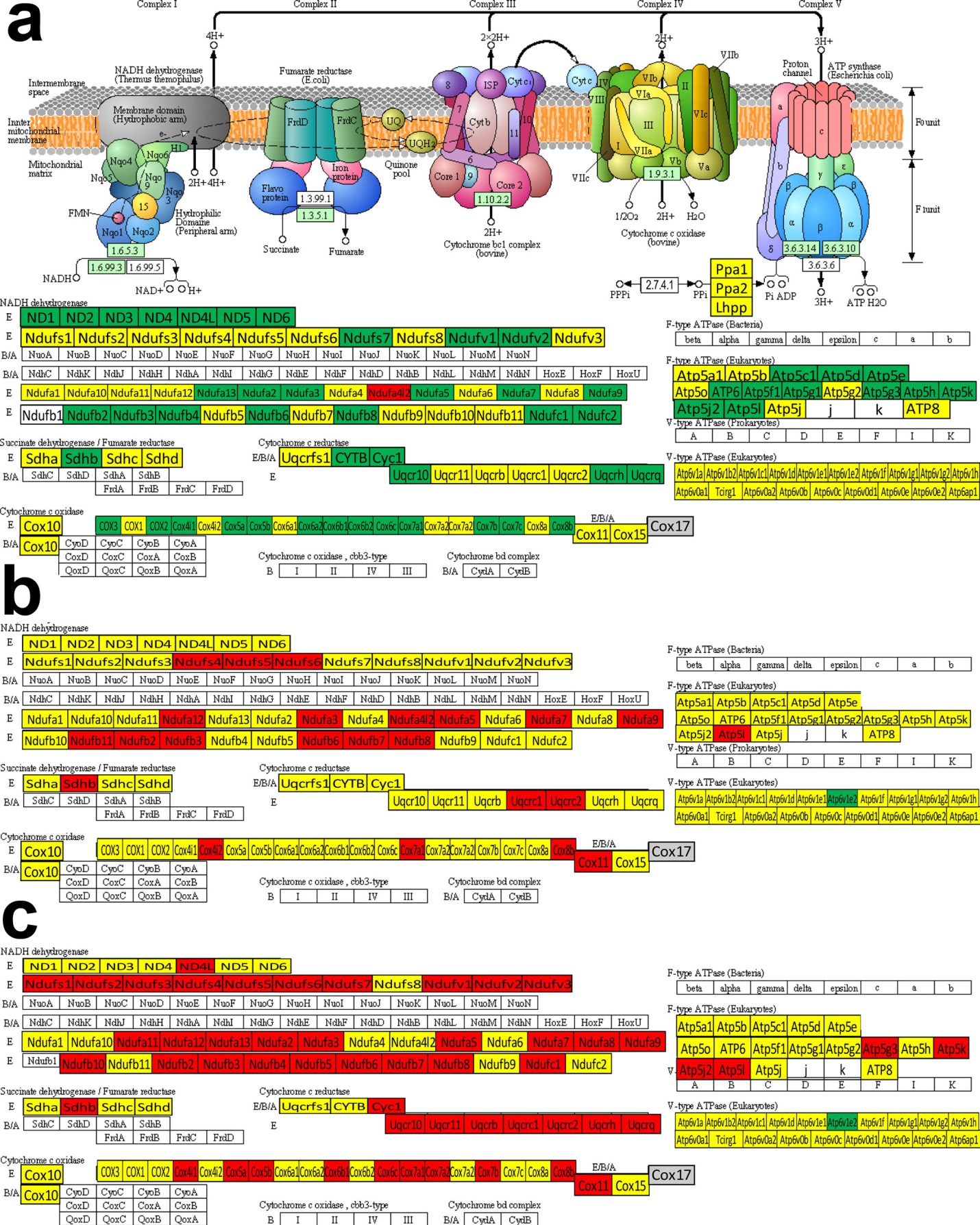
**

**Supplementary Figure 4: Expression correlation of *Ctnnb1* (catenin (cadherin associated protein), beta 1) with the potential partners of *Ank2.***

**
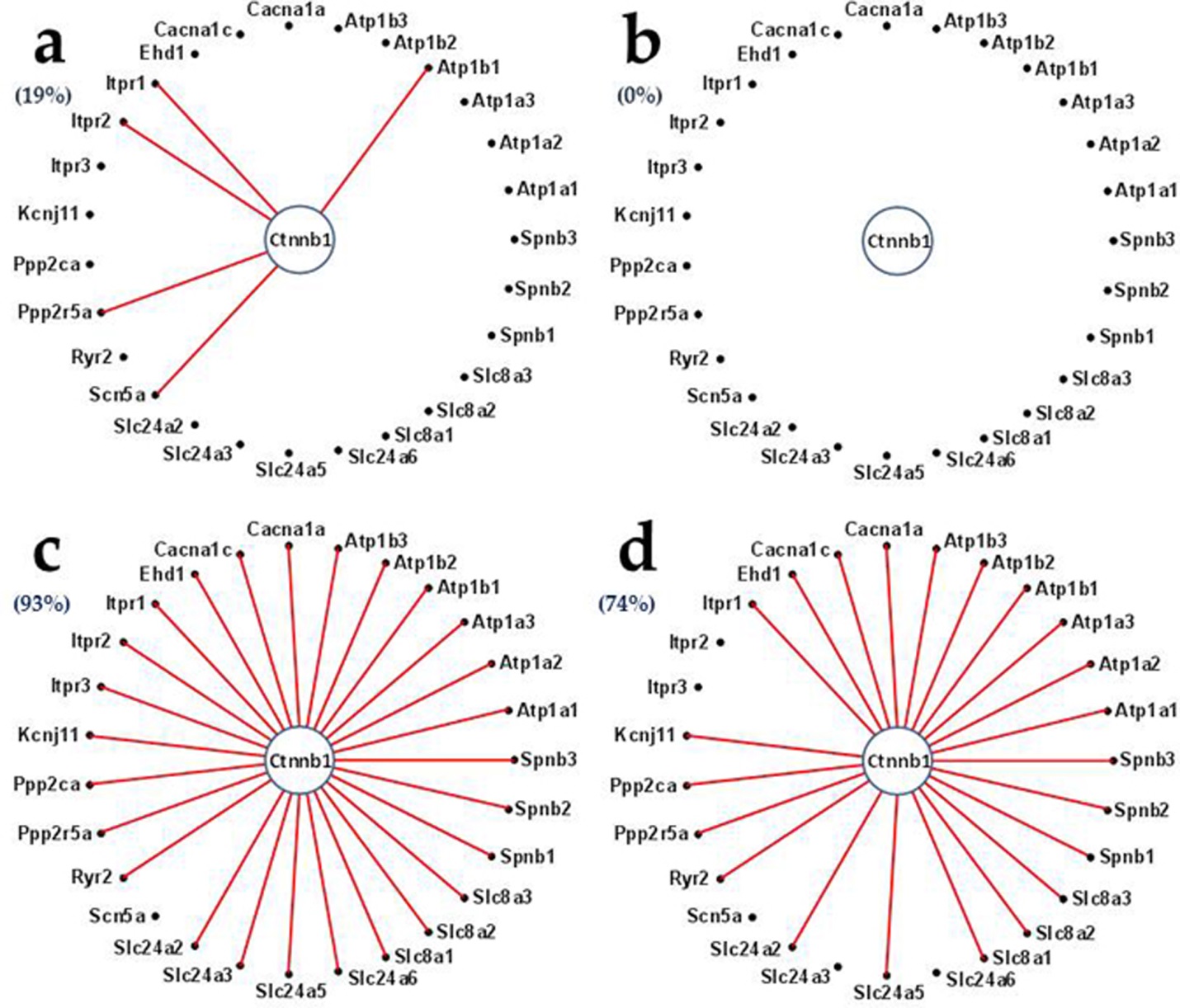
**

**Supplementary Figure 5: Expression correlation of *Hspa5* (heat shock protein 5) with the potential partners of *Ank2.***

***
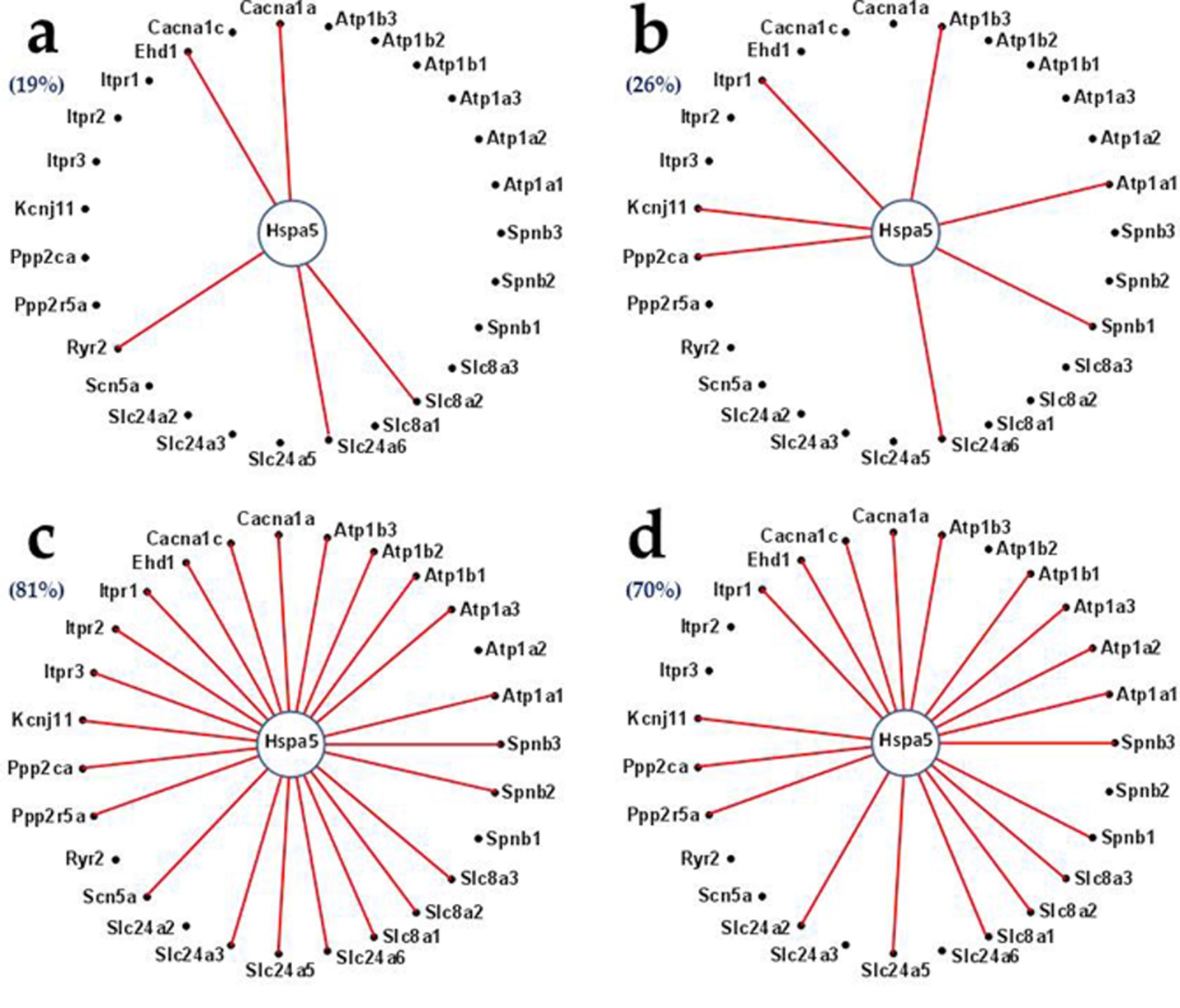
***

**Supplementary Figure 6: Expression correlation of *Mapk1* (mitogen-activated protein kinase 1)** **with the potential partners of *Ank2.***


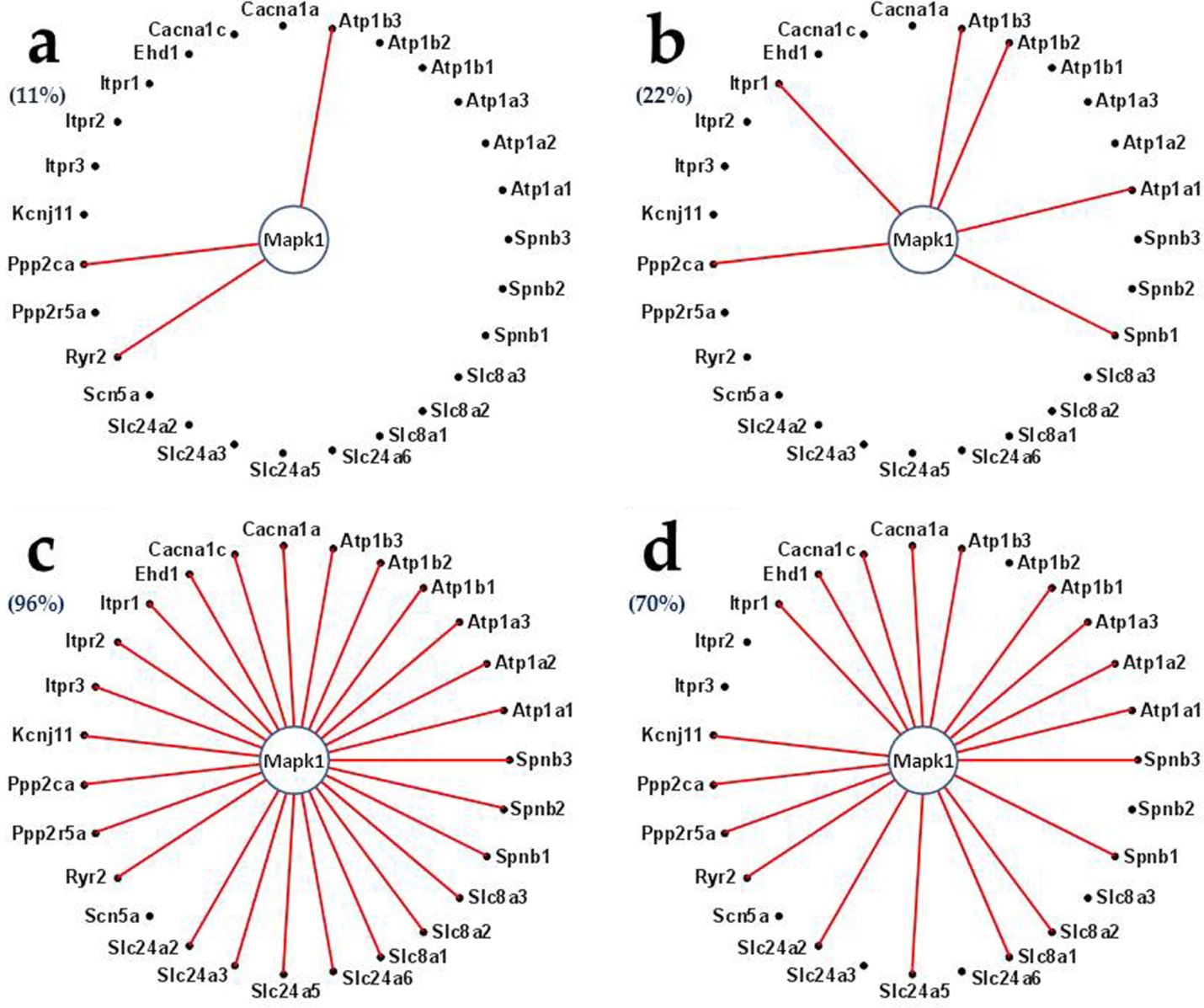

Supplement: Supplementary file 1 — Supplementary Information [file 41598_2021_82383_MOESM1_ESM.docx]
